# *CX3CR1^+^* Monocytes/Macrophages Promote Regional Immune Injury in Mesangial Proliferative Glomerulonephritis through Crosstalk with Activated Mesangial Cells

**DOI:** 10.34133/research.0716

**Published:** 2025-06-02

**Authors:** Jie Zhang, Qingyun Fang, Yiyu Huang, Yilun Qu, Qun Liu, Run Li, Yena Zhou, Shaoyuan Cui, Ran Liu, Xu Wang, Yunfeng Bai, Shuwei Duan, Lingling Wu, Pu Chen, Yong Wang, Jie Wu, Xuefeng Sun, Guangyan Cai, Ying Zheng, Quan Hong, Xiangmei Chen

**Affiliations:** ^1^ School of Medicine, Nankai University, Tianjin 300071, China.; ^2^ Department of Nephrology, First Medical Center of Chinese PLA General Hospital, State Key Laboratory of Kidney Diseases, National Clinical Research Center for Kidney Diseases, Beijing Key Laboratory of Medical Devices and Integrated Traditional Chinese and Western Drug Development for Severe Kidney Diseases, Beijing Key Laboratory of Digital Intelligent TCM for the Prevention and Treatment of Pan-vascular Diseases, Key Disciplines of National Administration of Traditional Chinese Medicine(zyyzdxk-2023310), Beijing 100853, China.; ^3^ School of Basic Medical Sciences, Chengdu University of Traditional Chinese Medicine, Chengdu 611137, China.

## Abstract

Mesangial proliferative glomerulonephritis (MsPGN) is the most common glomerulonephritis pathological type, including IgA nephropathy (IgAN), in which regional immune injury leads to disease progression without targeted treatment approaches. The mechanism of regional immune injury in MsPGN is unclear. We previously performed single-cell RNA sequencing (scRNA-seq) of IgAN and identified that the *CX3CR1* gene increased in kidney. In this study, further scRNA-seq analysis and cellchat analysis revealed that *CX3CL1* and *CX3CR1* expression was increased in mesangial cells and monocytes/macrophages, respectively, in IgAN, mediating stronger crosstalk. This result and its association with regional immune injury were validated in clinical specimens and MsPGN animal model. Deficiency of *CX3CR1^+^* monocytes/macrophages in the MsPGN animal model attenuated proteinuria, cell proliferation, and inflammation in glomerulus. Mechanistically, CX3CL1 in activated mesangial cells induced *CX3CR1^+^* monocyte/macrophage migration and activation, and RNA-seq, Luminex multiplex immunoassay, and molecular analysis revealed that *CX3CR1^+^* monocytes/macrophages induced mesangial cell injury via the MIF–CD74 interaction and activated the phosphatidylinositol 3-kinase (PI3K)/proteinserine-threonine kinase (AKT) pathway. Lastly, the therapeutic effect of the CX3CL1 monoclonal antibody quetmolimab was validated for inhibiting the progression of MsPGN. These findings demonstrate that activated mesangial cells interact with *CX3CR1^+^* monocytes/macrophages promoting glomerulus regional immune injury in MsPGN, providing evidence into the CX3CL1–CX3CR1 axis as a novel target of treatment for MsPGN.

## Introduction

Mesangial proliferative glomerulonephritis (MsPGN) is the most common pathological type of primary glomerulonephritis worldwide [1–3], including IgA nephropathy (IgAN). MsPGN is characterized by infiltration of inflammatory cells, mesangial cell proliferation, and increased extracellular matrix (ECM) [1–4]. The progression of MsPGN leads to glomerulus sclerosis, renal interstitial fibrosis, and end-stage renal disease (ESRD) eventually [5]. Its mechanism underlying disease pathogenesis and progression is still unclear, leading to the gap in satisfactory therapies. The traditional treatment focuses on the renin–angiotensin–aldosterone system (RAAS) inhibition and steroid-based immunosuppression in appropriate population [6,7]. In addition, therapeutic agents targeting B cells and plasma cells, such as rituximab and felzartamab, can effectively reduce the production of Gd-IgA1 in IgAN [8,9]. Furthermore, complement inhibitors targeting key components (e.g., factor B, C3, and C5) demonstrate considerable therapeutic potential. Notable examples include iptacopan, IONIS-FB-LRx, pegcetacoplan, and cemdisiran [10–12]. Thus, a better understanding of the mechanism of MsPGN pathogenesis and progression will help to initiate the development of the novel therapeutic targets.

Regional immune injury was caused by mutual influence of circulatory immune cells, resident immune cells, and renal intrinsic cells to maintain the immune environment in kidney disease [13]. Monocytes and macrophages play a key role in the regulation of renal inflammatory response [14–16], as a bond of circulatory immune and regional immune. Activated mesangial cells have been recognized as a key to the progression of MsPGN disease, which proliferate severely, produce ECM, secrete various inflammatory factors under disease conditions, and destroy glomerulus function [17–19]. An increasing number of studies have confirmed that activated mesangial cells play an immunomodulatory role interacting with immune cells in kidney disease [20]. There is still a large knowledge gap and space in mechanism of mesangial cell–monocyte/macrophage crosstalk and its function in the glomerulus regional immune injury of MsPGN.

Our previous study performed single-cell RNA sequencing (scRNA-seq) of IgAN patients. Results identified up-regulated *CX3CR1* gene in monocytes–macrophages of kidney [21,22]. CX3CR1, the exclusive G-protein-coupled receptor of CX3CL1, is most abundant in monocytes and macrophages [23], facilitating immune cell adhesion and recruitment [24,25]. CX3CL1 (also known as fractalkine) is the only member of the CX3C subfamily [26]. CX3CL1 is expressed in many types of cells in the kidney and regulated by proinflammatory and profibrotic cytokines [27,28], including mesangial cells [29,30]. In IgAN, CX3CL1 circulating level is up-regulated, associated with progression of kidney function and pathological damage and renal outcome [31]. These findings indicate that the CX3CL1–CX3CR1 interaction may involve in the pathogenesis and glomerulus regional immune injury of MsPGN. However, its exact mechanism and potential as a therapeutic target remain unclear.

In this study, we determined the increase and importance of *CX3CR1^+^* monocytes/macrophages in glomerulus and CX3CL1 expression in mesangial cells in IgAN patients and MsPGN animal model. Mechanically, activated mesangial cells secreted CX3CL1 and induced *CX3CR1^+^* monocyte/macrophage migration and activation. Reversely, activation of *CX3CR1^+^* monocyte-derived macrophages further damaged mesangial cells via the MIF/CD74/phosphatidylinositol 3-kinase (PI3K)/proteinserine-threonine kinase (AKT) pathway, looping cell crosstalk. Lastly, the therapeutic effect of the anti-CX3CL1 monoclonal antibody quetmolimab for inhibiting progression of MsPGN was validated. Our research provided a better understanding in the mechanism of activated mesangial cells interacting with *CX3CR1^+^* monocytes/macrophages and promoting regional immune injury in MsPGN, and offers valuable guidance on developing the CX3CL1–CX3CR1 axis as a potential novel therapeutic target.

## Results

### scRNA-seq analysis of *CX3CR1* and *CX3CL1* in kidney of IgAN patients

**Fig. 1.**
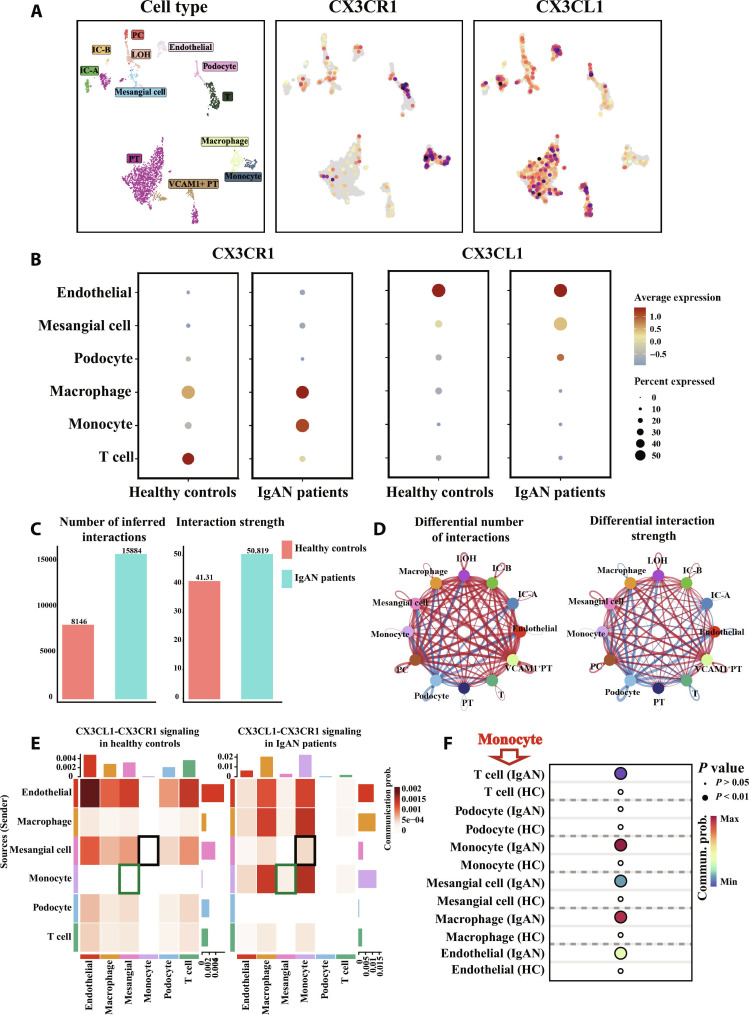
scRNA-seq analysis of *CX3CR1* and *CX3CL1* in kidney of IgAN patients. (A) Uniform manifold approximation and projection (UMAP) plot showing cell cluster identities and *CX3CR1* and *CX3CL1* abundance in kidney of IgAN patients. (B) Bubble plot showing differential *CX3CR1* and *CX3CL1* expression in immune cells and glomerulus intrinsic cells in kidney of healthy controls and IgAN patients. (C) Bar plot showing the number of inferred interactions and interaction strength between kidney cells in healthy controls and IgAN patients. (D) Visualized network graphs showing cell type-specific differential number of interactions and differential interaction strength among kidney of healthy controls and IgAN patients. Color in blue represents the down-regulation in IgAN, and red represents the up-regulation in IgAN. (E) Heatmap showing CX3CL1–CX3CR1 signaling between immune cells and glomerulus intrinsic cells in kidney of healthy controls and IgAN patients. Communication prob., communication probability. (F) Bubble plot showing differential CX3CL1–CX3CR1 signaling from monocytes to subcell types in kidney of healthy controls and IgAN patients. Two-tailed *t* test. PT, proximal tubular cells; VCAM1^+^PT, vascular cell adhesion molecule 1–positive proximal tubular cells; LOH, loop of helen; PC, principal cells; IC-A, intercalated cells A; IC-B, intercalated cells B.

To explore the abundance of *CX3CR1* and *CX3CL1* gene expression in all kinds of kidney cell types of MsPGN, we used previous scRNA-seq dataset of healthy controls and IgAN patients for further analysis. We identified 12 cell types in kidney and analyzed *CX3CR1* and *CX3CL1* expression in IgAN patients. The *CX3CR1* expression was significant on monocytes, macrophages, and T cells, with *CX3CL1* expressed on all kinds of intrinsic cells in kidney (Fig. 1A). Then, we analyzed differential *CX3CR1* and *CX3CL1* expression in glomerulus intrinsic cells and immune cells among healthy controls and IgAN patients (Fig. 1B). Between groups, *CX3CR1* expression in monocytes/macrophages and *CX3CL1* expression in mesangial cells was remarkably higher in the IgAN group. *CX3CR1* expression in T cells and *CX3CL1* expression in endothelial cells were also abundant, while there was no significant increase in IgAN compared with healthy controls. We suggested that the CX3CL1–CX3CR1 interaction between mesangial cells and monocytes/macrophages might be the key in glomerulus regional immune injury in IgAN. Intercellular crosstalk analysis revealed a significant increase in the number of inferred interactions and interaction strength in IgAN patients (Fig. 1C and D). Specially, CX3CL1–CX3CR1 signaling was analyzed in crosstalk between glomerulus intrinsic cells and immune cells. Consistent with our hypothesis, CX3CL1–CX3CR1 signaling was stronger between mesangial cells and monocytes in IgAN patients compared to healthy controls (Fig. 1E and F). These results indicated the involvement of CX3CR1 and CX3CL1 in IgAN, and the CX3CL1–CX3CR1 axis might mediate mesangial cell–monocyte/macrophage crosstalk participating in glomerulus regional immune injury in MsPGN.

### *CX3CR1^+^* monocytes/macrophages in glomerulus and CX3CL1 expression in mesangial cells were increased in MsPGN and correlated with MsPGN progression

**Fig. 2.**
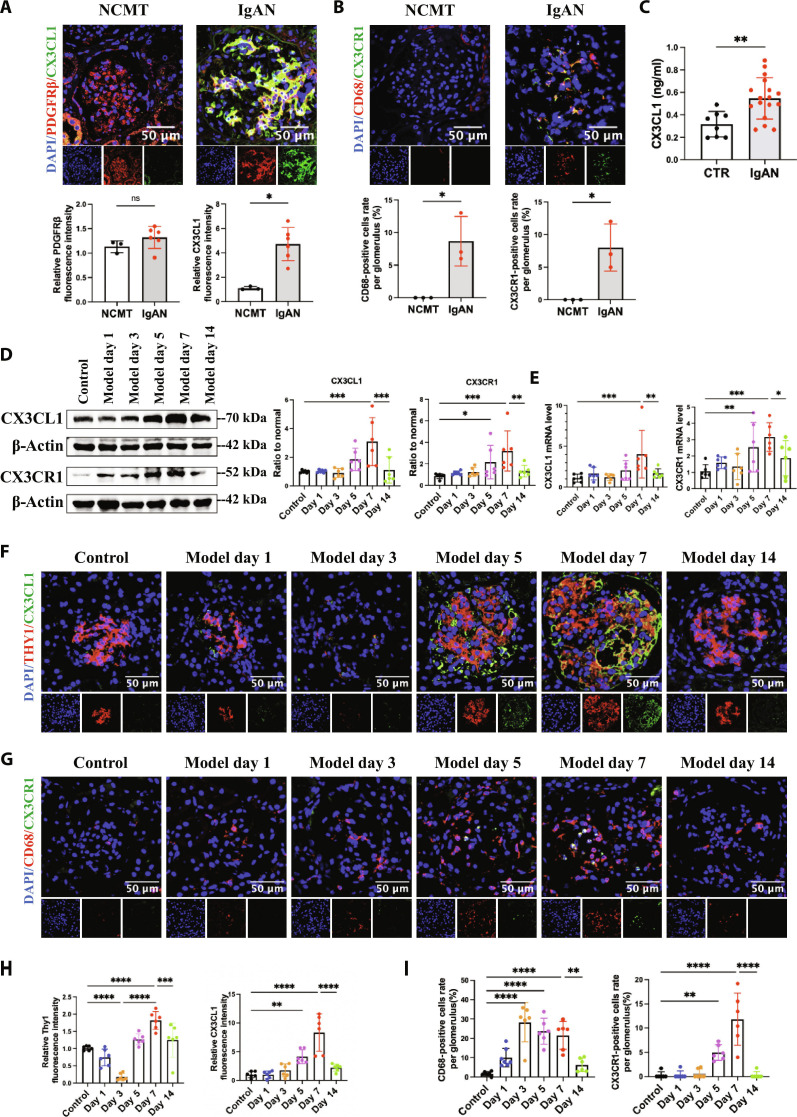
The expression and localization of CX3CL1 and CX3CR1 in glomerulus of IgA nephropathy patients and anti-Thy1 nephritis model. Immunofluorescence microscopy was performed on NCMT and kidney sections of IgAN patients. (A) CX3CL1 (green) is costained with PDGFRβ (red), and (B) CX3CR1 (green) is costained with CD68 (red). Scale bar, 50 μm. NCMT, *n* = 3. IgAN, *n* = 6. (C) Plasma CX3CL1 level was measured in IgAN patients (*n* = 17) and healthy controls (*n* = 8). (D) Western blot analysis of CX3CL1 and CX3CR1 protein in glomerulus of anti-Thy1 nephritis at different time points. (E) Real-time polymerase chain reaction (PCR) analysis of *CX3CL1* and *CX3CR1* mRNA expression in indicated groups. *n* = 6. Immunofluorescence microscopy was performed on kidney sections of anti-Thy1 nephritis at different time points. (F and H) CX3CL1 (green) is costained with THY1 (red), and (G and I) CX3CR1 (green) is costained with CD68 (red). Scale bar, 50 μm. *n* = 6. Results are presented as means ± SD. *P < 0.05; **P < 0.01; ***P < 0.001; ****P < 0.0001; ns, not significant.

To validate the result of scRNA-seq analysis, we collected kidney biopsy tissue of 6 IgAN patients (the clinical information is shown in Table) and blood samples of 8 healthy controls and 17 IgAN patients. Immunofluorescent staining showed overexpression of CX3CL1 and revealed colocalization with mesangial cell marker platelet-derived growth factor receptor β (PDGFRβ) in IgAN patients compared to noncancerous matched tissue (NCMT), with more *CD68^+^CX3CR1^+^* cells in the glomerulus (Fig. 2A and B). Meanwhile, the CX3CL1 expression was significantly increased in the plasma of IgAN patients (Fig. 2C).

**Table.** Clinical information of IgAN patients

Patient’s ID	Gender	Age	Diagnosis	Pathological diagnosis
392	Female	57	Chronic nephritis syndrome	IgAN (Lee’s grades III, Oxford Classification M0E0S0T1C0)
394	Female	34	Chronic nephritis syndrome	IgAN (Lee’s grades III~IV, Oxford Classification M1E0S1T1C0)
408	Female	35	Chronic nephritis syndrome	IgAN (Lee’s grades III, Oxford Classification M0E0S1T0C0)
409	Female	39	Chronic nephritis syndrome	IgAN (Lee’s grades III, Oxford Classification M0E0S1T0C0)
547	Female	67	Chronic nephritis syndrome	IgAN (Lee’s grades III, Oxford Classification M1E1S1T0C0)
550	Male	52	Chronic nephritis syndrome	IgAN (Lee’s grades III, Oxford Classification M0E0S1T1C0)

The anti-Thy1 nephritis model was established as the MsPGN model [32] to determine the regulation of CX3CL1 and CX3CR1 during the development of disease. We collected kidney tissue, glomerulus tissue, urine, and blood samples of rats on days 1, 3, 5, 7, and 14 after model establishment (Fig. S1A). Day 5 to day 7 was the mesangial proliferative stage with mesangial cell proliferation, ECM increase, and proteinuria (Fig. S1B to E). During this stage, CX3CL1 and CX3CR1 expression in glomerulus increased and peaked on day 7 (Fig. 2D and E). Meanwhile, the expression of mesangial cells marker in rats THY1 was elevated and costained with CX3CL1, with a large number of *CD68^+^* cells (monocytes/macrophages) infiltrating into glomerulus, in which a part was costained with CX3CR1 (Fig. 2F to I).

To explore CX3CL1 expression and secretion in activated mesangial cells in the condition of inflammatory and proliferation in vitro, we cultured human renal mesangial cells (HRMCs) with tumor necrosis factor-α (TNFα) (0 to 50 ng/ml) and PDGF-BB (0 to 100 ng/ml) stimulation [33]. The CX3CL1 expression in HRMCs increased with the increase of TNFα and PDGF-BB concentration until it reached 10 and 20 ng/ml, respectively (Fig. S2A). Immunofluorescent staining and enzyme-linked immunosorbent assay (ELISA) analysis showed that HRMCs expressed and secreted CX3CL1 markedly with TNFα (10 ng/ml) and PDGF-BB (20 ng/ml) (Fig. S2B and C). These results suggested that *CX3CR1^+^* monocytes/macrophages in glomerulus and up-regulated CX3CL1 expression in activated mesangial cells were associated with mesangial cell proliferation and the progression of regional immune inflammation in MsPGN.

### Deficiency of *CX3CR1^+^* monocytes/macrophages in glomerulus alleviated proteinuria, cell proliferation, and inflammation in MsPGN

**Fig. 3.**
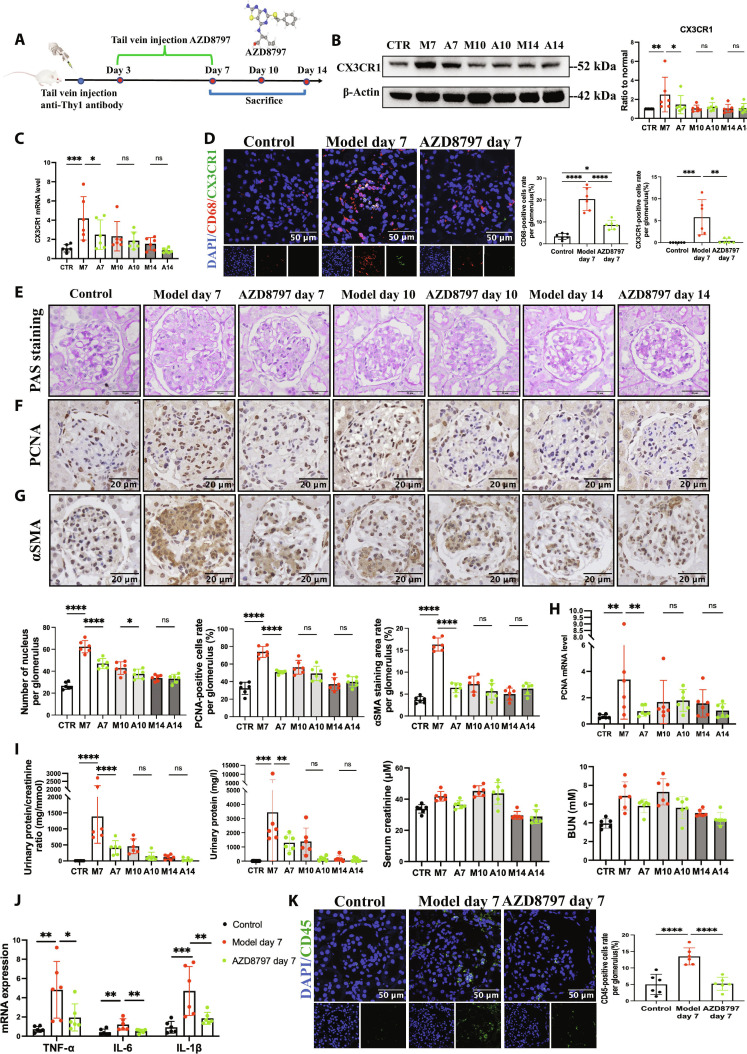
CX3CR1 antagonist AZD8797 alleviated proteinuria, cell proliferation, and inflammation in glomerulus of anti-Thy1 nephritis. (A) Experimental outline. Rats had injection of 2.5 mg/kg anti-Thy1 antibody via tail vein to establish anti-Thy1 nephritis model. CX3CR1 antagonist AZD8797 2 mg/kg or equal volume of vehicle control was injected daily on day 3 to 7 after model establishment. Rats were sacrificed on days 7, 10, and 14 after model establishment. (B) Western blot analysis of CX3CR1 protein level in glomerulus of indicated groups. CTR, control; M7, model day 7; A7, AZD8797 day 7; M10, model day 10; A10, AZD8797 day 10; M14, model day 14; A14, AZD8797 day 14; *n* = 6. (C) Real-time PCR analysis of *CX3CR1* mRNA expression in indicated groups. *n* = 6. Immunofluorescence microscopy was performed on kidney sections of indicated groups. (D) CX3CR1 (green) is costained with CD68 (red). Scale bar, 50 μm. *n* = 6. Representative images and quantitative results of PAS staining (E) and immunostaining for PCNA (F) and αSMA (G) in the glomerulus of indicated groups. Scale bar, 20 μm. *n* = 6. (H) Real-time PCR analysis of *PCNA* mRNA expression in indicated groups. *n* = 6. (I) The urinary albumin-to-creatinine ratio (UACR) was determined with the levels of urinary protein, serum creatinine, and BUN in indicated groups. *n* = 6. (J) Real-time PCR analysis of *TNFα*, *IL-6*, and *IL-1β* mRNA expression in indicated groups. *n* = 6. (K) Immunofluorescence microscopy detection on CD45 expression of indicated groups. Scale bar, 50 μm. *n* = 6. Results are presented as means ± SD. *P < 0.05; **P < 0.01; ***P < 0.001; ****P < 0.0001; ns, not significant.

We established anti-Thy1 nephritis and blocked the infiltration of *CX3CR1^+^* monocytes/macrophages into injured glomerulus by injecting AZD8797-CX3CR1 antagonist (Fig. 3A). The expression of CX3CR1 in glomerulus was attenuated the in AZD8797 group on day 7, but no difference on days 10 and 14 (Fig. 3B and C). *CX3CR1^+^* and total monocyte/macrophage infiltration were also observably reduced in glomerulus of the AZD8797 group on day 7 (Fig. 3D), indicating that AZD8797 effectively reduced *CX3CR1^+^* monocyte chemotaxis to glomerulus and differentiation into macrophages. Periodic acid–Schiff (PAS) staining and immunohistochemical results showed that AZD8797 attenuated the number of nucleated cells and PCNA^+^ cells, αSMA expression in the glomerulus, especially on day 7 (Fig. 3E to H). Biochemical measurements revealed that AZD8797 attenuated proteinuria of anti-Thy1 nephritis on day 7 with no influence on serum creatinine and blood urea nitrogen (BUN) level (Fig. 3I). *TNFα*, *IL-6*, and *IL-1β* mRNA expression with *CD45^+^* cell infiltration in glomerulus of the AZD8797 group on day 7 was decreased compared with the model group (Fig. 3J and K). These results indicated that AZD8797 inhibited *CX3CR1^+^* monocyte/macrophage infiltration, blocked the CX3CL1–CX3CR1 interaction, and ameliorated proteinuria, cell proliferation, cell activation, and inflammation in glomerulus of MsPGN. Given that AZD8797 also functions as an allosteric antagonist of CXCR2, we detected CXCR2 mRNA expression in glomerulus to exclude its potential involvement in the intervention effects of AZD8797. Results showed that CXCR2 mRNA expression increased in the model group but remained unchanged with AZD8797 treatment (Fig. S3), suggesting that the intervention of AZD8797 in MsPGN was primarily CX3CR1-mediated. This observation might be attributed to the relatively low CXCR2 expression in circulating monocytes, which was up-regulated in inflammatory tissues. By intravenous administration, AZD8797 primarily targeted CX3CR1 on peripheral blood monocytes.

### CX3CL1 in activated mesangial cells promoted *CX3CR1^+^* monocyte/macrophage migration and activation

**Fig. 4.**
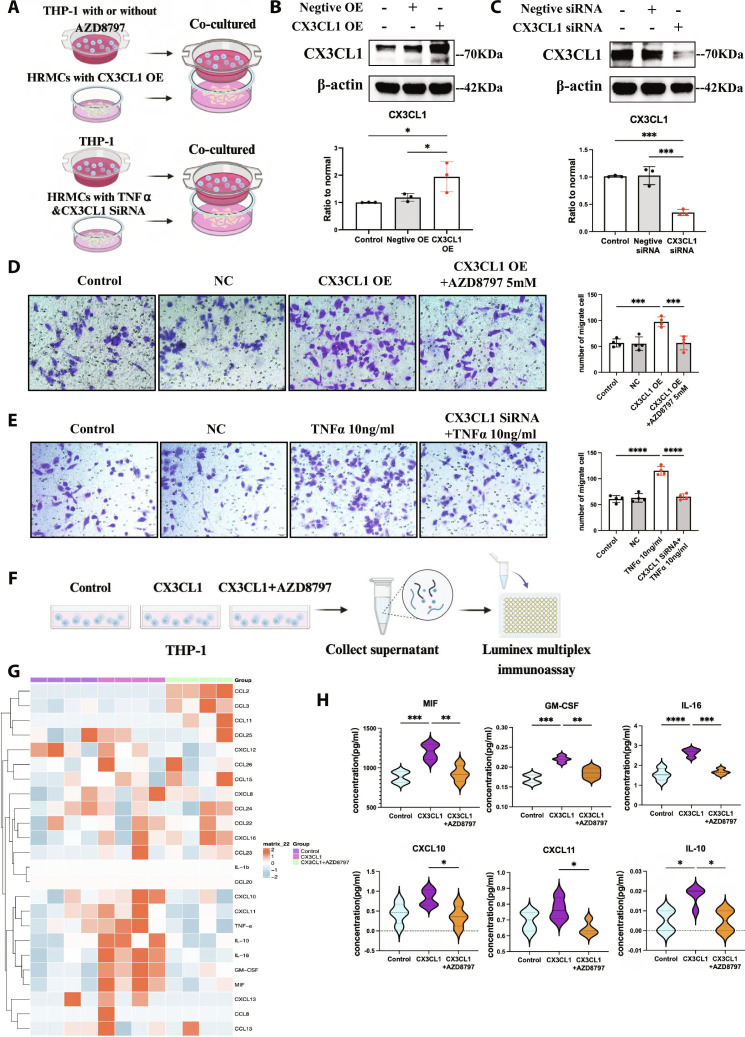
CX3CL1–CX3CR1 interaction mediated monocyte/macrophage migration and activation induced by mesangial cells. (A) THP-1 was treated with AZD8797 and cocultured with CX3CL1 overexpressed HRMCs in transwell system. HRMCs were transfected with CX3CL1 siRNA or not under TNFα stimulation and cocultured with THP-1 in transwell system. (B and C) Western blotting detects the transfection efficiency of CX3CL1 overexpression plasmid and CX3CL1 siRNA in HRMCs. *n* = 3. (D) Crystal violet staining of THP-1 cocultured with normal HRMCs, HRMC-transfected negative plasmid (NC), or CX3CL1 overexpression plasmid (OE) for 48 h, and the inhibition of THP-1 migration was assessed by CX3CR1 antagonist AZD8797 under the overexpressed CX3CL1. *n* = 4. Scale bar, 50 μm. (E) Crystal violet staining of THP-1 cocultured with normal HRMCs, HRMC-transfected negative siRNA (NC), HRMCs stimulated by TNFα (10 ng/ml), and HRMC-transfected CX3CL1 siRNA under 10 ng/ml TNFα stimulation for 48 h. *n* = 4. Scale bar, 50 μm. (F) The supernatant of THP-1 and THP-1 under 50 ng/ml CX3CL1 treatment with or without 5 mM AZD8797 for 48 h was collected for Luminex multiplex immunoassay. (G) Heatmap of 24 factors analyzed by Luminex multiplex immunoassay in indicated groups. *n* = 4. (H) Statistical analysis of cytokines with significant differences in indicated groups. Results are presented as means ± SD. *P < 0.05; **P < 0.01; ***P < 0.001; ****P < 0.0001; ns, not significant.

THP-1 is an established human immortalized monocyte-like cell line for in vitro study of monocyte differentiation and monocyte/macrophage function, expressing CX3CR1 constitutively [34,35]. As shown in Fig. S4A and B, THP-1 migration increased under the treatment of human recombinant CX3CL1 (0 to 100 ng/ml) and suppressed by CX3CR1 antagonist AZD8797 (0 to 10 mM). These results preliminary confirmed that CX3CL1–CX3CR1 promoted THP-1 migration. To verity this effect under the influence of activated mesangial cells, we cocultured THP-1 and HRMCs in transwell inserts and plates (Fig. 4A). We regulated CX3CL1 expression in HRMCs with transfecting overexpression plasmid or small interfering RNAs (siRNAs) (Fig. 4B and C). AZD8797 was used further to block the CX3CL1–CX3CR1 interaction between HRMCs and THP-1. Compared with normal HRMCs and HRMC-transfected negative plasmid, the migration of THP-1 cells through the insert membrane increased when cocultured with HRMC-transfected CX3CL1 overexpression plasmid. This effect was suppressed by AZD8797 (Fig. 4D). Besides, high CX3CL1 expression in HRMCs activated by TNFα induced more THP-1 migration, which was attenuated with the transfection of CX3CL1 siRNA (Fig. 4E).

On account of the correlation between CX3CL1 overexpression with more *CX3CR1^+^* monocytes/macrophages and glomerulus injury in MsPGN, we speculated that *CX3CR1^+^* monocytes/macrophages activated by CX3CL1 might cause stronger damage than the resting ones. We treated THP-1 with human recombinant CX3CL1 for 48 h (THP-1^CX3CL1^) in vitro, with or without blocked CX3CR1 by AZD8797, and cell supernatants were collected for Luminex multiplex immunoassay (Fig. 4F). Twenty-four factors related to immune effect were detected in cell supernatants (Fig. 4G and Table S2). We found that the concentration of cytokines among the groups had significant differences (Fig. 4H), including chemokines [CXCL10, CXCL11, interleukin-6 (IL-6)] and factors participating in inflammatory and immune responses [macrophage migration inhibitory factor (MIF), IL-10, and granulocyte-macrophage colony-stimulating factor (GM-CSF)]. It is suggested that *CX3CR1^+^* monocytes/macrophages activated by CX3CL1 had severe immunoregulation effect. Thereinto, MIF is a proinflammatory cytokine with multiple functions and participates in various inflammatory conditions [36]. GM-CSF is the member among the CSF group. It affects various immune cells and mediates cell survival, cell adhesion, cell differentiation, and immunomodulation [37]. IL-16 as a chemoattractant of T cells can stimulate the production of proinflammatory cytokines in various inflammatory diseases. Besides, CXCL10, CXCL11, and IL-10 also played critical roles in immune disease [38,39]. However, no significant differences in CXCL10 and CXCL11 levels were observed between the control and CX3CL1 groups. Notably, the levels of CCL2 and CCL3 increased in the AZD8797 group, while the CX3CL1 group maintained normal levels. This suggested that AZD8797 might activate certain effects in THP-1, which was also a disadvantage of using allosteric antagonists. Table S2 shows that while statistically significant differences were observed, the absolute levels of CCL2 and CCL3 remained remarkably low in indicated groups, making them unlikely to have biological relevance or interfere with experimental outcomes. However, these observations highlighted a critical pharmacological consideration for the application of allosteric antagonists.

### MIF–CD74 interaction mediated crosstalk between *CX3CR1^+^* monocytes/macrophages and mesangial cells

**Fig. 5.**
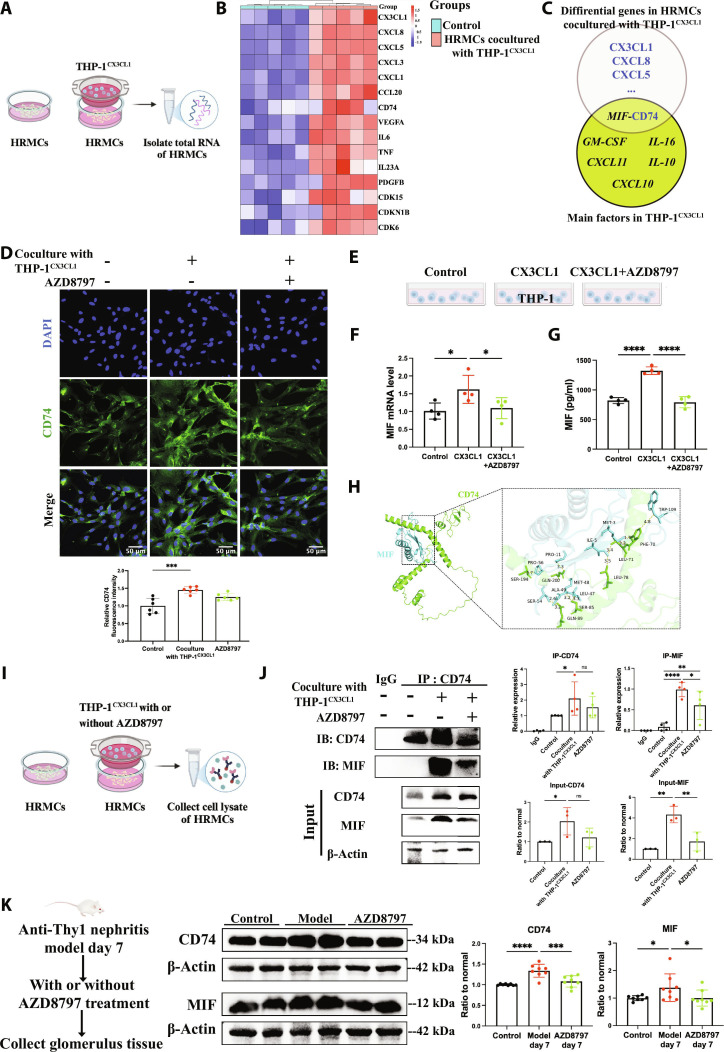
CX3CL1–CX3CR1 promoted the binding of MIF and CD74 between monocytes/macrophages and mesangial cells. (A) The total RNA of HRMCs cocultured with or without THP-1^CX3CL1^ was isolated for RNA-seq. (B) Cluster analysis of the differentially expressed genes identified by RNA-seq and related to chemotaxis of immune cells, inflammation, and cell proliferation in HRMCs cocultured with THP-1^CX3CL1^ for 48 h or not. (C) Interaction molecular pair between HRMCs cocultured with THP-1^CX3CL1^ and THP-1^CX3CL1^. (D) Immunofluorescence microscopy detection on CD74 expression in normal HRMCs and HRMCs cocultured with THP-1^CX3CL1^ for 48 h under 5 mM AZD8797 treatment or not. *n* = 6. Scale bar, 50 μm. (E to G) *MIF* mRNA expression and protein level in supernatant of THP-1 and THP-1 treated by 50 ng/ml CX3CL1 with or without 5 mM AZD8797 for 48 h. *n* = 4. (H) MIF was docked with CD74, and the binding site analysis was determined. (I and J) Co-IP analysis of MIF and CD74 within the indirect cell coculture system of HRMCs and THP-1. (K) Western blot analysis of CD74 and MIF expression in glomerulus of normal rats and anti-Thy1 nephritis on day 7 with or without AZD8797 treatment. *n* = 6. Results are presented as means ± SD. *P < 0.05; **P < 0.01; ***P < 0.001; ****P < 0.0001; ns, not significant.

To further explore the mechanism by which *CX3CR1^+^* monocyte/macrophage damage to mesangial cells, we isolated total RNA from HRMCs cocultured with or without THP-1^CX3CL1^ for RNA-seq (Fig. 5A). A total of 2544 genes were differentially expressed between 2 groups (|log FC| > 2 and *P* < 0.05). In the coculture with the THP-1^CX3CL1^ group, 1,133 genes were up-regulated with 1,411 genes were suppressed (Fig. S5A). Figure 5B shows different genes related to cell proliferation, inflammation, and chemotaxis of immune cells in indicated groups. Remarkably, *CX3CL1* expression was increased in HRMCs cocultured with THP-1^CX3CL1^, which lead to a CX3CL1–CX3CR1 interaction loop between HRMCs and THP-1. In addition, we found that the *CD74* gene was up-regulated in HRMCs cocultured with THP-1^CX3CL1^, which was reported as the MIF receptor in other studies [40] (Fig. 5C). Fluorescence staining result showed that CD74 expression was in HRMCs structurally and increased after coculture with THP-1^CX3CL1^ (Fig. 5D). There was no significant difference in the AZD8797 group. To further determine the Luminex multiplex immunoassay result of MIF in THP-1, we detected MIF expression and secretion, which was promoted by CX3CL1 and inhibited by AZD8797 (Fig. 5E to G). A visible molecular docking between MIF and CD74 was performed in HawkDOCK SERVER as previously reported [41]. Result showed that MIF had significant binding force with CD74 (Fig. 5H), and the binding site analysis showed that the binding free energy of the complex was −59.27 (kcal/mol). Additionally, the functional interaction between MIF and CD74 was further confirmed by co-immunoprecipitation (Co-IP) assay using the endogenous proteins from HRMCs cocultured with or without THP-1^CX3CL1^ for 48 h compared with the AZD8797 group (Fig. 5I). Co-IP analysis showed that MIF secreted by THP-1 had the potential to interact with CD74 in HRMCs. The MIF–CD74 interaction was promoted in coculture with THP-1^CX3CL1^ and suppressed in the AZD8797 group (Fig. 5J). The CD74 protein level in the AZD8797 group in IP analysis and input analysis has no significant difference to the THP-1^CX3CL1^ group. This might be because CD74 up-regulation is mediated via other pathways. We also determined the CD74 and MIF expression in glomerulus of the MsPGN animal model. Results showed that CD74 and MIF were up-regulated in glomerulus of anti-Thy1 nephritis on day 7 and decreased by AZD8797 (Fig. 5K). These findings indicated that the CX3CL1–CX3CR1 axis promoted the MIF–CD74 interaction between monocytes/macrophages and mesangial cells.

### MIF/CD74/PI3K/AKT pathway mediated *CX3CR1^+^* monocytes/macrophages-induced mesangial cell injury

**Fig. 6.**
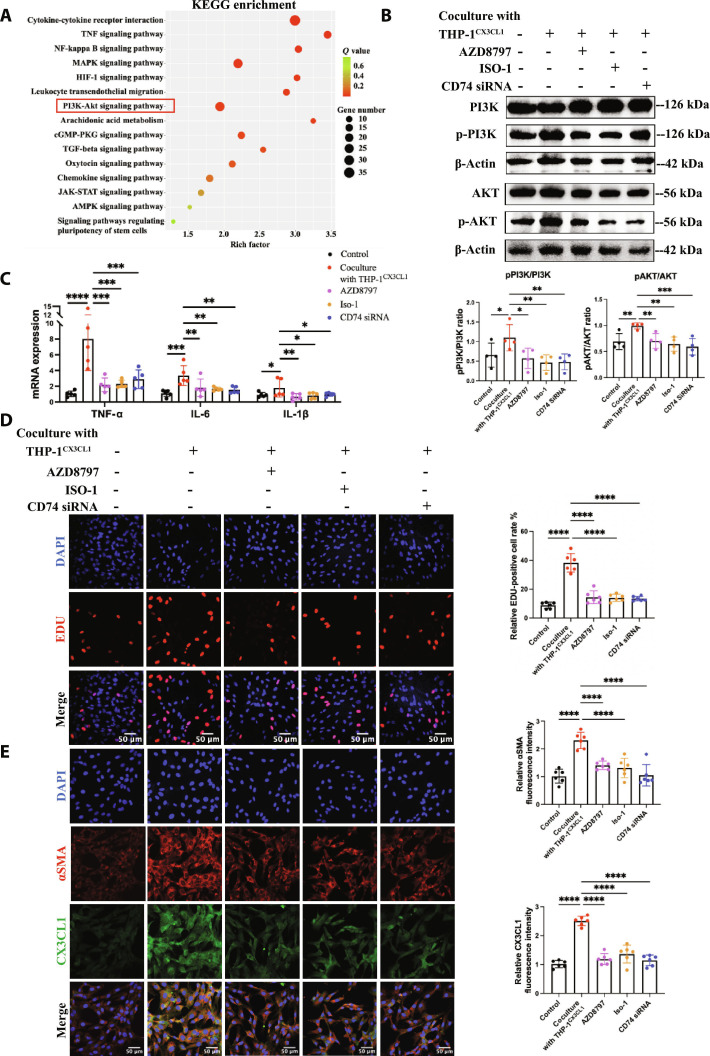
CX3CL1–CX3CR1 mediated monocyte-induced mesangial cell injury via the MIF/CD74/PI3K/AKT pathway. (A) KEGG enrichment shows the PI3K–AKT signaling pathway. (B) Western blot analysis of pAKT/AKT level, pPI3K/PI3K level in normal HRMCs, HRMCs cocultured with THP-1^CX3CL1^ for 48 h treated or not with AZD8797 and ISO-1, and CD74 knockdown HRMCs cocultured with THP-1^CX3CL1^ for 48 h. *n* = 4. (C) Real-time PCR analysis of *TNFα*, *IL-6*, and *IL-1β* mRNA expression in normal HRMCs, HRMCs cocultured with THP-1^CX3CL1^ for 48 h treated or not with AZD8797 and ISO-1, and CD74 knockdown HRMCs cocultured with THP-1^CX3CL1^ for 48 h. *n* = 5. (D) EDU was performed to detect cell proliferation of normal HRMCs, HRMCs cocultured with THP-1^CX3CL1^ for 48 h treated or not with AZD8797 and ISO-1, and CD74 knockdown HRMCs cocultured with THP-1^CX3CL1^ for 48 h. *n* = 6. Scale bar, 50 μm. (E) Immunofluorescence microscopy detection on αSMA (red) and CX3CL1 (green) expression of normal HRMCs, HRMCs cocultured with THP-1^CX3CL1^ for 48 h treated or not with AZD8797 and ISO-1, and CD74 knockdown HRMCs cocultured with THP-1^CX3CL1^ for 48 h. *n* = 6. Scale bar, 50 μm. Results are presented as means ± SD. *P < 0.05; **P < 0.01; ***P < 0.001; ****P < 0.0001; ns, not significant.

The Kyoto Encyclopedia of Genes and Genomes (KEGG) enrichment analysis of RNA-seq in HRMCs revealed that the signaling pathways were notably enriched in the PI3K–AKT signaling pathway, cytokine–cytokine receptor interaction, cell adhesion molecules, the mitogen-activated protein kinase (MAPK) signaling pathway, and so on (Fig. 6A). Gene Ontology (GO) enrichment analysis was shown in Fig. S5B. Based on previous studies, the PI3K–AKT signaling pathway could be activated by MIF–CD74 in other disease [42]. We speculated that CX3CL1 expressed in mesangial cells might promote *CX3CR1^+^* monocytes/macrophages secreting MIF, which bind to CD74 in mesangial cells and activated the PI3K–AKT pathway. To validate this hypothesis, we suppressed the MIF–CD74 interaction by CD74 siRNA (Fig. S5C) and MIF inhibitor ISO-1. Then, we analyzed the expression and phosphorylation of AKT and PI3K in HRMCs cocultured with THP-1^CX3CL1^, which were increased compared to the control group, AZD8797 group, MIF inhibitor ISO-1 group, and CD74 knockdown group (Fig. 6B and Fig. S5D).

The mRNA expression of *TNFα*, *IL-6*, and *IL-1β* in HRMCs cocultured with THP-1^CX3CL1^ was higher than the other groups (Fig. 6C). Meanwhile, the 5-ethynyl deoxyuridine (EDU) assay result showed that the EDU^+^ cells were increased in HRMCs cocultured with THP-1^CX3CL1^, which were decreased in the AZD8797 group, MIF inhibitor ISO-1 group, and CD74 knockdown group (Fig. 6D). The expression of αSMA and CX3CL1 in HRMCs cocultured with THP-1^CX3CL1^ was also suppressed by blocking the CX3CL1–CX3CR1 and MIF–CD74 interaction (Fig. 6E). These results revealed that *CX3CR1^+^* monocytes/macrophages activated by CX3CL1 promoted mesangial cell proliferation, activation, and inflammation via the MIF/CD74/PI3K/AKT pathway. Remarkably, the MIF–CD74 interaction between monocytes/macrophages and mesangial cells further promoted the expression of CX3CL1 in mesangial cells, resulting in the further cell interaction and injury cycle repeat.

**Fig. 7.**
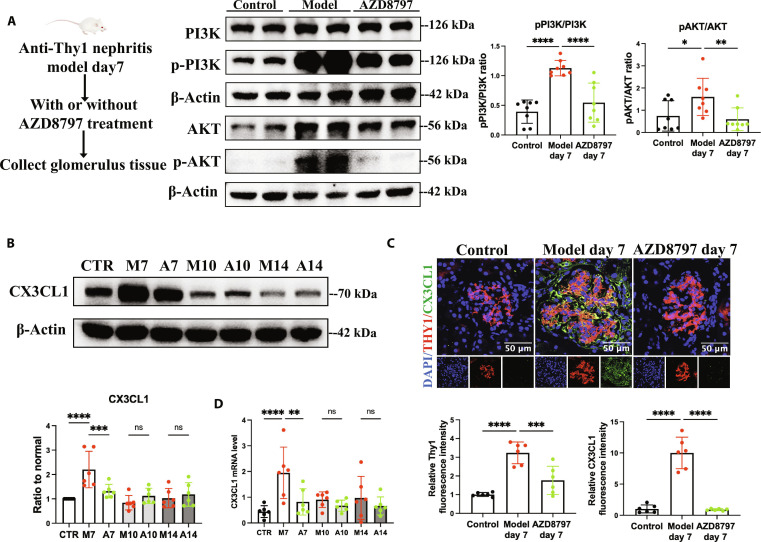
CX3CR1 antagonist AZD8797 inhibited the PI3K/AKT pathway and suppressed CX3CL1 expression in glomerulus of anti-Thy1 nephritis. (A) Western blot analysis of pAKT/AKT level, pPI3K/PI3K level in glomerulus of normal rats, and anti-Thy1 nephritis on day 7 with or without AZD8797 treatment. *n* = 6. (B) Western blot analysis of CX3CL1 protein level in glomerulus of indicated groups. CTR, control; M7, model day 7; A7, AZD8797 day 7; M10, model day 10; A10, AZD8797 day 10; M14, model day 14; A14, AZD8797 day 14; *n* = 6. Immunofluorescence microscopy was performed on kidney sections of indicated groups. (C) CX3CL1 (green) is costained with THY1 (red). Scale bar, 50 μm. *n* = 6. (D) Real-time PCR analysis of *CX3CL1* mRNA expression in indicated groups. *n* = 6. Results are presented as means ± SD. *P < 0.05; **P < 0.01; ***P < 0.001; ****P < 0.0001; ns, not significant.

In vivo, MIF–CD74 expression and PI3K–AKT phosphorylation were decreased in glomerulus of anti-Thy1 nephritis in the AZD8797 group on day 7 (Fig. 7A and Fig. S6). It provided the evidence for the MIF/CD74/PI3K/AKT pathway activated by CX3CL1–CX3CR1 in vivo. With such effect in the glomerulus, the CX3CL1 expression in mesangial cells was also reduced (Fig. 7B to D) due to the decreased inflammation and inhibited the MIF/CD74/PI3K/AKT pathway.

### Anti-CX3CL1 monoclonal antibody quetmolimab ameliorated glomerulus regional immune injury in MsPGN

**Fig. 8.**
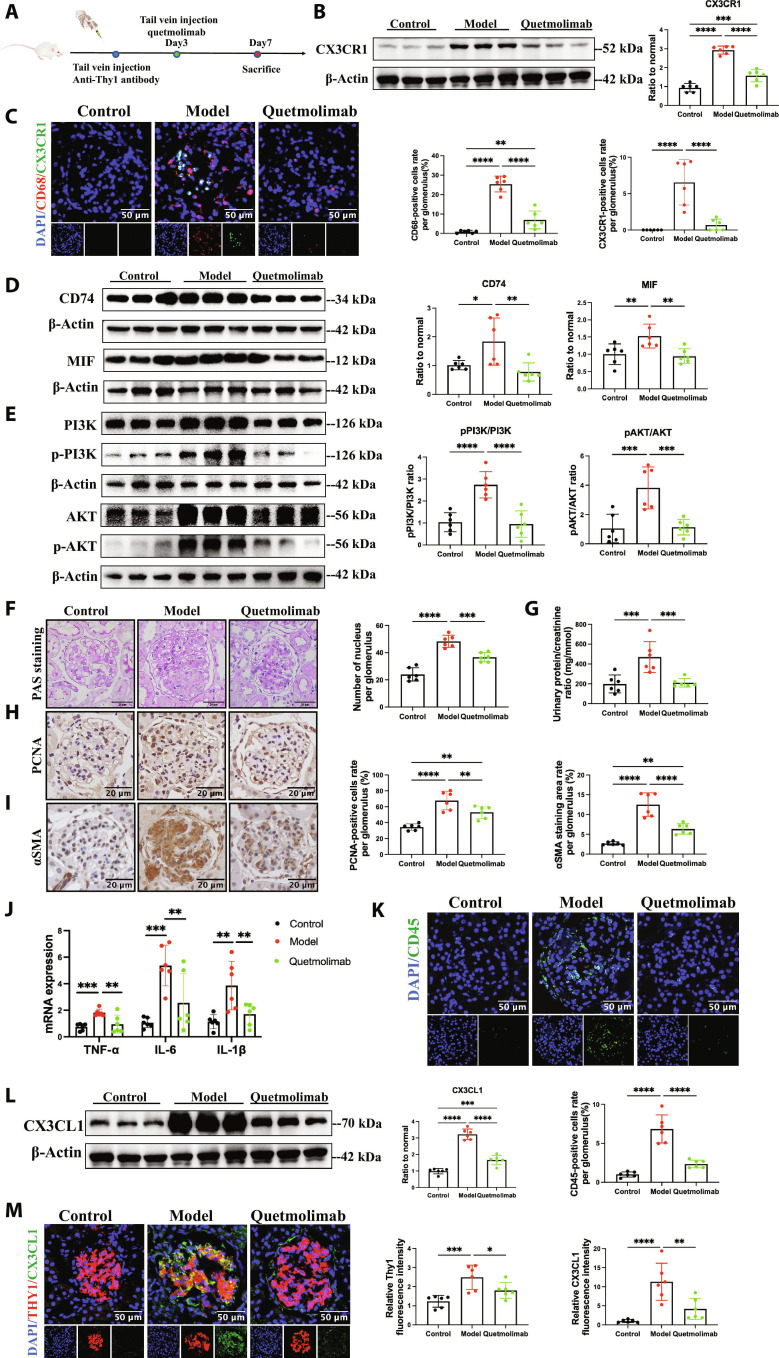
Quetmolimab decreased kidney injury and inflammation in anti-Thy1 nephritis, inhibiting the MIF/CD74/PI3K/AKT pathway. (A) Experimental outline. Rats had injection of 2.5 mg/kg anti-Thy1 antibody via tail vein to establish anti-Thy1 nephritis model. Then, rats were injected of quetmolimab 3 mg/kg or equal volume of vehicle control on day 3 after model establishment and sacrificed on day 7 after model establishment. (B) Western blot analysis of CX3CR1 protein level in glomerulus of indicated groups. *n* = 6. Immunofluorescence microscopy was performed on kidney sections of indicated groups. (C) CX3CR1 (green) is costained with CD68 (red). Scale bar, 50 μm. *n* = 6. (D and E) Western blot analysis of CD74 and MIF expression, pAKT/AKT level, and pPI3K/PI3K level in glomerulus of normal rats and anti-Thy1 nephritis on day 7 with or without quetmolimab treatment. *n* = 6. (G) UACR was determined in indicated groups. *n* = 6. Representative images and quantitative results of PAS staining (F) and immunostaining for PCNA (H) and αSMA (I) in the glomerulus of indicated groups. Scale bar, 20 μm. *n* = 6. (J) Real-time PCR analysis of *TNFα*, *IL-6*, and *IL-1β* mRNA expression in indicated groups. *n* = 6. (K) Immunofluorescence microscopy detection on CD45 expression of indicated groups. Scale bar, 50 μm. *n* = 6. (L) Western blot analysis of CX3CL1 protein level in glomerulus of indicated groups. *n* = 6. Immunofluorescence microscopy was performed on kidney sections of indicated groups. (M) CX3CL1 (green) is costained with THY1 (red). Scale bar, 50 μm. *n* = 6. Results are presented as means ± SD. *P < 0.05; **P < 0.01; ***P < 0.001; ****P < 0.0001; ns, not significant.

To explore potential clinical translational value of our findings, we verified the therapeutic effect of quetmolimab in the MsPGN model. Quetmolimab, a humanized anti-CX3CL1 monoclonal antibody, is undergoing clinical trials for the treatment of various inflammatory diseases, including rheumatoid arthritis and Crohn disease [43,44]. We established the anti-Thy1 nephritis model and injected quetmolimab through the tail vein on day 3, the initiation of proliferative phase (Fig. 8A). The expression of CX3CR1 and infiltration of *CX3CR1^+^* monocytes/macrophages in the glomerulus of the quetmolimab group were significantly reduced (Fig. 8B and C and Fig. S7A), suggesting that quetmolimab effectively neutralized CX3CL1 and inhibited its chemotactic effect on *CX3CR1^+^* monocytes. Meanwhile, the MIF/CD74/PI3K/AKT pathway was also inhibited in the quetmolimab group (Fig. 8D and E and Fig. S7G). As a consequence, quetmolimab attenuated proteinuria, cell proliferation, and activation in glomerulus (Fig. 8F to I and Fig. S7C and F). Then, quetmolimab suppressed *TNFα*, *IL-6*, and *IL-1β* mRNA expression and *CD45^+^* immune cell infiltration in glomerulus (Fig. 8J and K).

In order to evaluate the effect of quetmolimab on total immune environment in glomerulus, we isolated kidney glomerulus and prepared single-cell suspension for flow cytometry analysis. The results showed that quetmolimab significantly reduced *CD45^+^CD68^+^CX3CR1^+^* (*CX3CR1^+^* monocytes/macrophages), *CD45^+^CX3CR1^+^* (*CX3CR1^+^* immune cells), and *CD45^+^CD68^+^CX3CR1^−^* (*CX3CR1^−^* monocytes/macrophages) cell populations in glomerulus, with no significant difference in *CD45^+^CX3CR1^−^* (*CX3CR1^−^* immune cells) (Fig. S7D and E). It suggested that quetmolimab not only suppressed the chemotaxis of *CX3CR1^+^* monocytes but also further attenuated the recruitment of other immune cells. It may be related to the local production of more chemokines after *CX3CR1^+^* monocytes/macrophages were activated. Lastly, CX3CL1 expression in mesangial cells was reduced (Fig. 8L and M and Fig. S7B). These findings provided evidence for the MsPGN treatment with quetmolimab by inhibiting activated mesangial cell–*CX3CR1^+^* monocyte/macrophage crosstalk, but further clinical trials were needed to determine its efficacy and safety for MsPGN patients.

## Discussion

Glomerulus regional immune injury has always been a key problem in the progression of MsPGN. Due to its unclear mechanism of pathogenesis and complex immune microenvironment in the glomerulus, MsPGN is still in the absence of specific therapy [45]. In this study, we analyzed the involvement of the CX3CL1–CX3CR1 axis in IgAN patients and the MsPGN model and explored the mechanism of cell crosstalk in HRMCs and THP-1 coculture system. Lastly, we evaluated the therapeutic effect of quetmolimab. We demonstrated the mechanism of activated mesangial cell*–CX3CR1^+^* monocyte/macrophage crosstalk promoting glomerulus regional immune injury in MsPGN for the first time, and verified the therapeutic effect of blocking the CX3CL1–CX3CR1 axis in MsPGN.

The glomerulus microenvironment comprises interconnected cells that communicate via direct contact or molecular signaling, collectively maintaining critical functions of kidney. While prior researches have extensively explored glomerulus cell interactions, for instance, bidirectional cytokine diffusion across the basement membrane facilitates signal transduction between endothelial cells and podocytes [46]. Additionally, studies have identified the vascular endothelial growth factor A (VEGFA)/VEGFR2 and Angpt2/Tie2 pathways as key mediators of endothelial cell–mesangial cell communication in MsPGN [47]. Beyond intrinsic renal cells, interactions with immune cells significantly contribute to disease pathogenesis. Emerging evidence highlights that podocytes modulate macrophage polarization via epigenetic mechanisms to promote injury in membranous nephropathy [48], while histone-modifying enzymes in activated endothelial cells exacerbate renal fibrosis through macrophage recruitment [48]. In MsPGN, mesangial cells act as primary drivers of disease progression, whereas monocytes/macrophages serve as pivotal mediators of glomerulus regional immune injury. Our study specifically interrogated the mesangial cell–monocyte/macrophage crosstalk, aiming to explore its unique mechanism and identify novel therapeutic targets for MsPGN.

A growing body of evidence indicates that the CX3CL1–CX3CR1 axis mediated important events in the kidney disease progression [49]. Our previous study found the up-regulated *CX3CR1* gene expression in monocytes–macrophages in the kidney of IgAN patients by scRNA-seq [22]. This finding provided evidence that the CX3CL1–CX3CR1 axis might be the key point to glomerulus regional immune injury. In this study, the dataset of kidney of IgAN patients was further analyzed, profiling *CX3CL1* and *CX3CR1* gene expression cell types among IgAN patients and healthy controls. *CX3CL1* was structurally expressed in a variety of renal intrinsic cells and especially increased in mesangial cells in IgAN. We speculated that it might be related to mesangial cell proliferation in IgAN. *CX3CR1* expression was abundant in monocytes, macrophages, and T cells. Furthermore, previous studies have demonstrated that during the early stages of disease injury, adaptive immune responses, particularly *CD8^+^* T cells, are predominantly localized around the glomerulus [50]. Based on these observations, we proposed that monocytes/macrophages play a more critical role in the pathogenesis of MsPGN. Our previous research traced the origin of glomerulus macrophages and identified circulating monocytes as the primary source, but not resident [21]. Here, we determined that CX3CL1 expression in mesangial cells and *CX3CR1^+^* monocyte/macrophage infiltration in glomerulus was up-regulated and was associated with glomerulus regional immune injury in clinical specimen and anti-Thy1 nephritis. Consistent with prior studies, our data showed that activated mesangial cells under inflammatory/proliferative conditions up-regulated CX3CL1 [30,51], creating a gradient that drove monocyte migration via CX3CR1. Thus, we hypothesized that under pathological conditions, circulating *CX3CR1^+^* monocytes migrate into the glomerulus, where they differentiate into macrophages and contribute to regional immune injury. This study highlights the important role of *CX3CR1^+^* monocytes/macrophages in MsPGN, but it is important to note that the involvement of other immune cell receptors in disease cannot be ruled out. For example, the relative importance of CX3CR1 versus CCR2 may depend on disease stage and microenvironmental changes; other receptors, including CCR5 and CXCR4, may be associated in chronic phases. The complete receptor participation profile deserves further study.

Given the pivotal role of the CX3CL1–CX3CR1 axis in mediating mesangial cell–monocyte/macrophage crosstalk, we sought to identify its downstream inflammatory effectors. Notably, MIF emerged as a prime candidate. MIF is a pro-inflammatory factor with multiple functions and involved in different inflammatory conditions. Based on such character, inhibition of MIF is considered as a potential therapy for the therapeutic strategy of immune inflammatory diseases [36,52]. In the kidney, interventions to inhibit MIF ameliorated the development of IgAN, crescentic glomerulonephritis, and lupus nephritis in animal studies [53–56]. In addition, MIF also has proliferative effect, which is partly mediated by its receptor CD74 [57]. Our results indicated that CX3CL1 promoted circulating monocyte migration into glomerulus and MIF secretion in *CX3CR1^+^* monocyte-derived macrophages, with the expression of its receptor CD74 in mesangial cells. This suggested MIF–CD74 as a mediator of *CX3CR1^+^* monocyte/macrophage and mesangial cell crosstalk. MIF–CD74 activated pathways such as extracellular signal–regulated kinase (ERK), PI3K–AKT, nuclear factor κB (NFκB), and adenosine 5ʹ-monophosphate-activated protein kinase (AMPK) pathways [40,58]. We detected differential genes in HRMCs cocultured with THP-1^CX3CL1^ by RNA-seq. Analysis showed that the PI3K–AKT pathway was activated, which was involved in oxidative stress, inflammation, cell proliferation, cell apoptosis, and epithelial–mesenchymal transformation (EMT) [59]. We confirmed that *CX3CR1^+^* monocytes/macrophages promoted mesangial cell proliferation, activation, and inflammation through the MIF/CD74/PI3K/AKT pathway. Interestingly, we found that the THP-1 activated by CX3CL1 further promoted CX3CL1 expression in HRMCs, forming the interaction loop. Here, we found that the CX3CL1–CX3CR1 axis represents a novel upstream regulator of MIF–CD74 signaling, demonstrating remarkable specificity in mediating monocyte/macrophage–mesangial cell crosstalk. Unlike conventional pathways (e.g., NFκB and MAPK) that broadly regulate inflammatory responses, this newly identified pathway exhibits distinct selectivity for MsPGN pathogenesis. Importantly, therapeutic targeting of the CX3CL1/CX3CR1/MIF/CD74 pathway offers significant advantages by maintaining systemic inflammatory homeostasis while specifically inhibiting glomerulus injury, thereby presenting a safer alternative to pan-inflammatory inhibitors.

Cederblad et al. [60] found that AZD8797, as a noncompetitive allosteric regulator of CX3CR1, could block CX3CL1–CX3CR1 axis-mediated cell adhesion and downstream G-protein-mediated signaling pathways. AZD8797 was used in animal studies about multiple sclerosis and liver regeneration to verify the involvement of *CX3CR1^+^* immune cells in disease [61–63]. To suppress the infiltration of *CX3CR1^+^* monocytes/macrophages in the glomerulus in vivo, we injected AZD8797 to the MsPGN animal model via tail vein and blocked the CX3CL1–CX3CR1 interaction. Then, glomerulus regional immune injury was relived with decreased proteinuria, cell proliferation, activation, and inflammation in glomerulus. In vitro, AZD8797 inhibited THP-1 migration and activation under high CX3CL1 expression in HRMCs. Meanwhile, AZD8797 blocked the MIF/CD74/PI3K/AKT pathway in THP-1-HRMCs coculture system and the MsPGN animal model, followed by a decrease of CX3CL1 expression in mesangial cells. These findings collectively underscore the contribution of the CX3CL1–CX3CR1 axis and *CX3CR1^+^* monocytes/macrophages to the progression of MsPGN.

The CX3CL1–CX3CR1 axis is composed of unique ligand/receptor pairs, making it an attractive therapeutic target for immune diseases [64–66]. Recently, many small-molecule inhibitors and monoclonal antibodies targeting the CX3CL1–CX3CR1 axis have been developed [49]. Quetmolimab (E6011), a humanized monoclonal antibody targeting CX3CL1, has demonstrated favorable safety and preliminary therapeutic efficacy in multiple clinical trials of immune inflammatory diseases. Matsuoka et al. [44] conducted a multicenter, dose-escalation phase 1 study evaluating quetmolimab in 28 Crohn’s disease (CD) patients. Over 12 weeks, 3 serious adverse events occurred (2 CD progression, one anemia). Pharmacokinetics showed dose-proportional serum drug levels, reaching steady state by 4 to 6 weeks, with a 40% clinical response rate at 12 weeks [44]. In a randomized, double-blind trial, Tanaka et al. [67] tested quetmolimab (200 to 400 mg, biweekly) in 169 methotrexate-refractory rheumatoid arthritis patients over 104 weeks. The study demonstrated favorable safety and tolerability, with no serious adverse events reported [67,68]. Hiroko Tabuchi et al. [69] conducted a randomized, double-blind, placebo-controlled, single-dose escalation study to evaluate the safety, tolerability, and pharmacokinetics of quetmolimab (0.0006 to 10 mg/kg) in healthy adult men (*n* = 64). Clinical and animal studies of quetmolimab for MsPGN have not been conducted. In this study, we administered a single dose of 3 mg/kg quetmolimab when the MsPGN animal model entered the mesangial proliferation stage to evaluate the therapeutics. Our results showed that quetmolimab intercepted the CX3CL1–CX3CR1 interaction and mesangial cell–monocyte/macrophage crosstalk. Concurrently, quetmolimab was efficient in reducing proteinuria, cell proliferation, activation and inflammatory response, and immune cell infiltration in glomerulus. However, our study aims to establish proof-of-concept for the therapeutic potential of quetmolimab in MsPGN. Further animal studies and clinical trials will be required to obtain pharmacological data on its application in MsPGN, thereby more scientifically validating its safety and efficacy profile. Based on these evidences, we highlight the potential for therapies targeting the CX3CL1–CX3CR1 axis to benefit MsPGN patients.

In conclusion, we demonstrate the mechanism of activated mesangial cell–*CX3CR1^+^* monocyte/macrophage crosstalk promoting glomerulus regional immune injury in MsPGN for the first time. CX3CL1 in activated mesangial cells promoted *CX3CR1^+^* monocyte/macrophage migration and activation, and *CX3CR1^+^* monocytes/macrophages induced mesangial cell injury through the MIF/CD74/PI3K/AKT pathway. Our findings provided evidence into the CX3CL1–CX3CR1 axis as a novel target of treatment for MsPGN and quetmolimab has potential to be a candidate therapy.

## Materials and Methods

### scRNA-seq analysis

The scRNA-seq dataset (GSE127136) of IgAN was accessed on Gene Expression Omnibus (GEO) database and analyzed by Seurat R package (version 4.0.0), including normalization, scaling, gene expression visualization, and uniform manifold approximation and projection (UMAP) dimension reduction. The information of enrolled samples has been previously reported. We screened 100 to 20% of UMAP from the mitochondrial genome and selected highly variable genes for principal components analysis (PCA) and the top 15 significant principal components for UMAP dimensionality reduction and gene expression visualization. The cell types were annotated based on the known typical marker genes and differentially expressed genes (DEGs) calculated using the FindAllMarker function, using the default parameters provided by Seurat. We used CellChat R package to explore intercellular crosstalk networks. The cellular communication networks and signaling pathways of each ligand–receptor pair were summarized and visualized by using network diagram, bubble diagram, and heatmap.

### Human study

Human study was conducted in accordance with the Declaration of Helsinki and Chinese PLA General Hospital ethics board approval (no. S2022-388-01). All human samples were collected with informed consent. We collected kidney sections from 6 IgAN renal biopsy tissues and 3 para-cancer tissues. Blood from 17 IgAN patients and 8 controls was collected for detection of plasma CX3CL1 level.

### Animals and treatments

Wild-type, male, 6- to 8-week-old, 200- to 250-g Wistar rats were purchased from Speifu Laboratory Animal Corporation (Beijing, China). All rats were fed in constant temperature (20 °C), humidity (70%), and alternating day and night cycles. To investigate the regulation of CX3CL1/CX3CR1 expression during the development of anti-Thy1 nephritis, we injected anti-Thy1 antibody (2.5 mg/kg) via tail vein to establish the MsPGN model, while the control group was injected with equal volume of saline solution. To evaluate the CX3CL1–CX3CR1 involvement in anti-Thy1 nephritis, CX3CR1 antagonist AZD8797 was injected (2 mg/kg/day) by tail vein injection daily from days 3 to 7 after model establishment, while both control and model groups were given the same amount of vehicle. The rats were anesthetized with pentobarbital before sacrifice on days 7, 10, and 14 after model establishment to collect blood, urine, and kidney tissues. To evaluate the therapeutic effect of quetmolimab to anti-Thy1 nephritis, the quetmolimab group was given with quetmolimab solution (3 mg/kg) by tail vein injection on day 3 after model establishment, while both control and model groups were given the same amount of vehicle. All experiments in this study were performed according to the Guide for the Care and Use of Laboratory Animals (National Research Council of the USA, 1996) and approved by the Animal Ethics Committee of the Chinese PLA General Hospital (no. 2022-X18-30).

### Statistical analysis

The data analyses in this study were performed using GraphPad (version 8.0; GraphPad). The results were expressed as means ± SD. The normally distributed datasets were analyzed using the unpaired Student’s *t* test for 2-group comparisons, and one-way analysis of variance (ANOVA) followed by Tukey’s post hoc test for multiple comparisons among ≥3 groups. For all statistical comparisons, *P* < 0.05 was considered statistically significant.

## Supplementary Material

20250602-1

## Data Availability

The datasets analyzed in this study are obtained from the NCBI GEO database with search number GSE127136.
