# Erratum to “*CX3CR1*^+^ Monocytes/Macrophages Promote Regional Immune Injury in Mesangial Proliferative Glomerulonephritis through Crosstalk with Activated Mesangial Cells”

**DOI:** 10.34133/research.1194

**Published:** 2026-03-18

**Authors:** Jie Zhang, Qingyun Fang, Yiyu Huang, Yilun Qu, Qun Liu, Run Li, Yena Zhou, Shaoyuan Cui, Ran Liu, Xu Wang, Yunfeng Bai, Shuwei Duan, Lingling Wu, Pu Chen, Yong Wang, Jie Wu, Xuefeng Sun, Guangyan Cai, Ying Zheng, Quan Hong, Xiangmei Chen

**Affiliations:** ^1^ School of Medicine, Nankai University, Tianjin 300071, China.; ^2^ Department of Nephrology, First Medical Center of Chinese PLA General Hospital, State Key Laboratory of Kidney Diseases, National Clinical Research Center for Kidney Diseases, Beijing Key Laboratory of Medical Devices and Integrated Traditional Chinese and Western Drug Development for Severe Kidney Diseases, Beijing Key Laboratory of Digital Intelligent TCM for the Prevention and Treatment of Pan-vascular Diseases, Key Disciplines of National Administration of Traditional Chinese Medicine(zyyzdxk-2023310), Beijing 100853, China.; ^3^ School of Basic Medical Sciences, Chengdu University of Traditional Chinese Medicine, Chengdu 611137, China.

In the Research Article entitled “*CX3CR1*+ Monocytes/Macrophages Promote Regional Immune Injury in Mesangial Proliferative Glomerulonephritis through Crosstalk with Activated Mesangial Cells” [1], the authors have identified an error in Fig. 4. Due to an inadvertent error during the image assembly process, the incorrect image was presented for the HRMC-transfected negative plasmid (NC) group in Fig. 4, panel D.

**Fig. 4.**
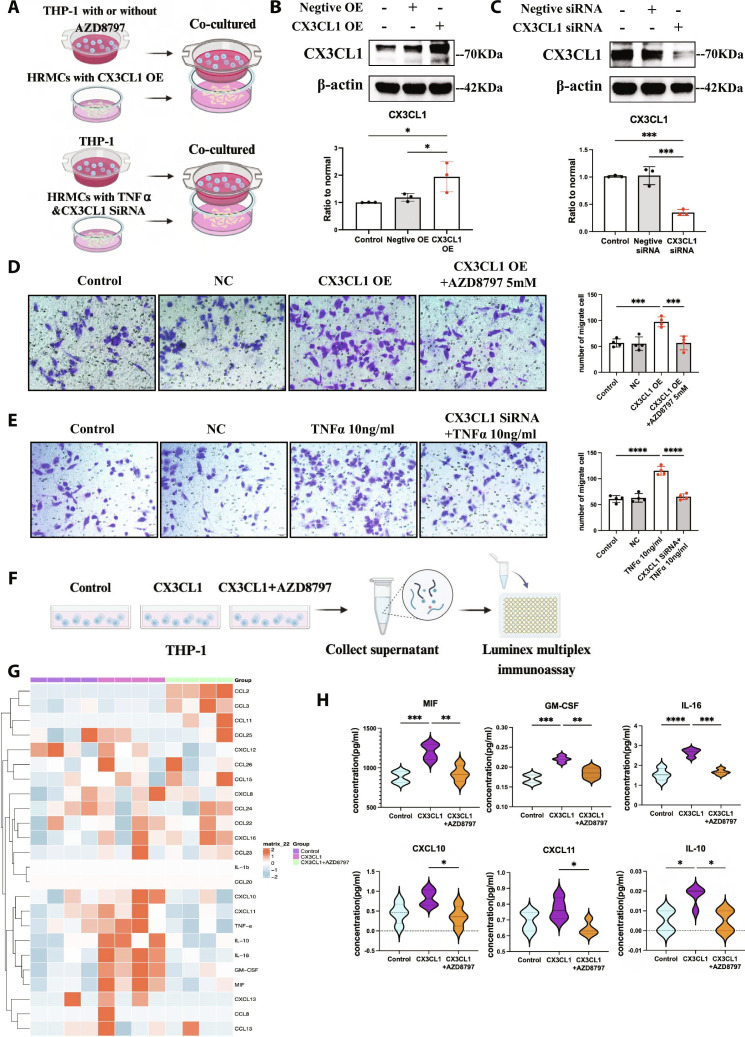
CX3CL1-CX3CR1 interaction mediated monocytes/macrophages migration and activation induced by mesangial cells. (A) THP-1 was treated with AZD8797 and co-cultured with CX3CL1 overexpressed HRMCs in transwell system. And HRMCs were transfected CX3CL1 siRNA or not under TNFα stimulation and co-cultured with THP-1 in transwell system. (B and C) Western blotting detects the transfection efficiency of CX3CL1 overexpression plasmid and CX3CL1 siRNA in HRMCs. *n* = 3. (D) Crystal violet staining of THP-1 co-cultured with normal HRMCs, HRMCs transfected negative plasmid (NC) or CX3CL1 overexpression plasmid (OE) for 48 hours, and assess the inhibition of THP-1 migration by CX3CR1 antagonist AZD8797 under the overexpressed CX3CL1. *n* = 4. Scale bar, 50μm. (E) Crystal violet staining of THP-1 co-cultured with normal HRMCs, HRMCs transfected negative siRNA (NC), HRMCs stimulated by TNFα (10ng/ml), HRMCs transfected CX3CL1 siRNA under 10ng/ml TNFα stimulation for 48 hours. *n* = 4. Scale bar, 50μm. (F) The supernatant of THP-1, THP-1 under 50 ng/mL CX3CL1 treatment with or without 5mM AZD8797 for 48 h was collected for Luminex multiplex immunoassay. (G) Heat map of 24 factors analyzed by Luminex multiplex immunoassay in indicated groups. *n* = 4. (H) Statistical analysis of cytokines with significant differences in indicated groups. Results are presented as the means ± SD, *P < 0.05; ** *P* < 0.01; ***P < 0.001; ****P < 0.0001; ns, not significant.

The authors apologize for this error and assure readers that this error does not affect the scientific conclusions drawn in the study. The corrected figure is presented below and has been corrected in the PDF and HTML versions of the article.
