# Supplementary material for: CX3CR1+ Monocytes/Macrophages Promote Regional Immune Injury in Mesangial Proliferative Glomerulonephritis through Crosstalk with Activated Mesangial Cells
Source: Research (Wash D C). 2025 Jun 2;8:0716. doi: 10.34133/research.0716 (PMC12128197; doi:10.34133/research.0716)
Supplement: Supplementary 1 — Supplementary Methods Tables S1 and S2 Figs. S1 to S7 [file research.0716.f1.pdf]

Supplementary Materials

Supplementary methods

Biochemical measurements

The urine obtained was centrifugated (1000g for 30 minutes at normal temperature) and stored at −80°C. The blood was collected in vacutainer tubes and centrifugated (3000rpm for 5 minutes at normal temperature). Then, the serum samples were collected and stored at −80°C. To evaluate the creatinine in urine and blood by enzymatic method for assaying, we used Creatinine Assay Kit (C011-2-1, Nanjing Jiancheng Bioengineering Institute). And urine albumin was measured by CBB method using Urine Protein Test Kit (C035-2-1, Nanjing Jiancheng Bioengineering Institute). Urine albumin creatinine ratio (UACR, mg/mmol) was calculated by urine albumin/creatinine. BUN in blood was detected by urease method using BUN Test Kit (C013-2-1, Nanjing Jiancheng Bioengineering Institute).

Periodic acid-schiff (PAS) staining

Rat kidneys were fixed in 10% formalin and dehydrated with Gradient ethanol. The tissue was embedded in paraffin and sectioned into 2 μm slices. Sections were stained with PAS and sealed with resin to assess glomerular cell proliferation. Twenty glomeruli were selected for each section.

Immunohistochemical staining

Paraffin kidney sections (2μm in thickness) were dewaxed with xylene and ethanol, incubated in 3% hydrogen peroxide and heated in a microwave to expose the antigen. The sections were blocked with goat serum (ZLI-9056, ORIGENE, CN) and incubated with primary antibodies (PCNA, 1:1000, ab92552, Abcam; αSMA, 1:100, ab7817, Abcam) overnight at 4°C. The sections were incubated

with the horseradish peroxidase streptavidin biotinylated secondary antibody followed by diaminobenzidine (DAB kit, Vector Laboratories, California, US).

Immunofluorescence staining

Kidney tissue cryosections (4μm in thickness) were fixed in 4 % paraformaldehyde, blocked with goat serum (ZLI-9056, ORIGENE, CN), and immuno-stained with primary antibodies (CX3CL1, 1:200, ab25088, Abcam; CX3CR1, 1:200, PA1-28839, ThermoFisher; CD45, 1:100, ab10558, Abcam; CD68, 1:100, ab31630, Abcam; Thy1, 1:100, self-produced; PDGFRβ, 1:100, ab69506, Abcam; αSMA, 1:100, ab7817, Abcam; CD74, 1:100, ab270265, Abcam). Secondary antibodies were FITC- or CY3-conjugated. Cell nuclei were visualized by 4', 6-diamidino-2-phenylindole (DAPI) staining. Slides were viewed under a confocal inverted laser microscope (FV3000, Olympus, Tokyo, Japan).

Flow cytometry

5 rats in each group were randomly selected to extract the kidney and remove the capsule completely. Cortical tissue was cut away from the medulla and minced in isolation buffer solution. Glomeruli were isolated by sequential sieving and collected， then had incubation in isolation buffer solution with collagenase IV (0.1%, MCE, HY-E70005D) and Liberase and DNase (0.25 mg/mL, 100 U/mL, Roche Applied Science, Indianapolis, IN, USA) for 30 minutes at 37°C. After centrifugation, cells were resuspended in PBS/1 % FBS, and filtered by 40 μm cell strainer for flow cytometric analysis using PE-CD68, FITC-CX3CR1, and PE/Cyanine7-CD45 antibodies (Bio-Legend). Cells were treated with fixation and permeabilization reagents using BD Cytofix / CytopermÔ Plus Fixation/Permeabilization Kit (BD Bioscience) according to the manufacturer's instructions. Flow cytometry analysis was performed on a DX flex (Beckman coulter). Data were analyzed using

cyexpert software.

Western blotting

Glomerulus tissue or cells were lysed with radioimmunoprecipitation assay (RIPA) lysis buffer containing protease inhibitors (1 μg/mL leupeptin, 1 μg/mL aprotinin and 100 μmol/L PMSF). After a 30 minutes incubation, the samples were centrifuged at 12000 g and 4°C for 30 minutes. The protein concentration was determined by a Pierce BCA protein assay kit (23225, ThermoFisher Scientific, MA, USA). Equal amounts of protein from each sample were separated by 10%-15% SDS-PAGE and then transferred onto nitrocellulose filter membranes. The membranes were blocked and incubated in antibodies against CX3CL1 (1:1000, ab25088, Abcam), CX3CR1 (1:1000, PA1-28839, ThermoFisher), CD74 (1:1000, ab64772, Abcam), MIF (1:1000, ab175189, Abcam), pAKT (1:1000, 13038S, CST), AKT (1:1000, 9272S, CST), pPI3K (1:1000, AF3242, Affinity), PI3K (1:1000, CST, 4257T) and β-actin (1:10000, 66009-1, Proteintech) overnight at 4°C. Finally, the membranes were incubated with secondary antibody at room temperature for 2 hours. Antigen-antibody complexes on the membranes were detected with an enhanced chemiluminescence kit from Thermo Scientific.

Quantitative real-time polymerase chain reaction (qRT-PCR)

Total RNA was extracted from glomerulus tissue and cells using the TRIzol RNA isolation system (Life Technologies, Grand Island, NY), and its concentration was quantified with a microspectrophotometer (Thermo Fisher Scientific, Inc., MA, USA). Total RNA (1ug) was reverse transcribed to cDNA utilizing a ProtoScript® II first strand cDNA synthesis kit (E6560S, New England Biolabs, Frankfurt, Germany), followed by quantitative PCR performed on an ABI Prism 7000 Sequence Detection System (Applied Biosystems, Foster City, CA, USA). The sequences of

primer pairs for various genes are detailed in Table S1. Data were normalized according to the level of 18s expression, and the relative expression of genes was generated with the comparative threshold cycle (Delta Ct) method.

Cell culture

Primary human renal mesangial cells (HRMCs) were purchased from ScienCell Research Laboratories (cat. 4200) and cultured in mesangial cell medium (cat. 4201) supplemented with 10% fetal bovine serum (FBS) (cat. 0010), 5 mL of MC growth factor (cat. 4252) and 5 mL of penicillin/streptomycin (P/S) solution (cat. 0503) at 37 ◦C and 5% CO2 in the cell incubator. HRMCs was treated with human recombination TNFα (0-50 ng/mL, Peprotech, cat.300–01a) and PDGF-BB (0-100 ng/mL, Peprotech, cat.100–14b). The human monocytes cell line THP-1 were purchased from the China Science Academy (Shang hai, China， SCSP-567) and cultured in RPMI-1640 meduim (Gibco, Grand Island, NY, USA) supplemented with 10%FBS, 100 U/mL of P/S and 0.05mM β mercaptoethanolat 37 °C and 5% CO2 in the cell incubator.

Cell co-culture

HRMCs were seeded into a 6-well plate or 24-well plate and cultured alone or stimulated with TNFα (10 ng/mL, Peprotech, cat.300–01a) or transfected siRNA and plasmid for 48h. THP-1 were initially cultured in 8um or 0.4um pore size transwell inserts (Corning, West Chester, PA) alone or stimulated with human recombination CX3CL1(50ng/ml, HY-P72685, MCE) for 48 h and were then washed with fresh media followed by co- culturing with HMCs for another 48h. AZD8797 (5mM, HY-13848, MCE)was added to the coculture system when indicated.

Cell migration assay

The transwell inserts carried THP-1 were washed by PBS and fixed in 4 % paraformaldehyde. Then

cells were stained by Crystal violet dye (C0121-100ml, Beyotime) and observed to assess cells migration. Data were collected from 4 independent experimental repeats.

Co-IP

Co-IP was performed by Co-Immunoprecipitation Kit (PK10007, Proteintech) according to manufacturer's instructions. The cell lysates were incubated with the indicated antibodies (CD74, 2ug/ml, ab64772, Abcam) or respective IgG overnight at 4 °C.

Small interfering (si) RNA transfection

HRMCs were seeded into a 6-well plate. When HRMCs reached 60%-70% confluence, the cells were transfected with 75pmol per well CX3CL1 siRNA, CD74 siRNA, or negative control using siRNA Transfection Reagent (GP-transfect-Mate, GenePharma) according to manufacturer's instructions.

Plasmid transfection

HRMCs were seeded into a 6-well plate. When HRMCs reached 80% confluence, jetPRIME in vitro DNA&siRNA Transfection Reagent (101000046, Polyplus-transfection S.A, Illkirch, France) was used for transfection CX3CL1 plasmid according to manufacturer's instructions.

Cell Proliferation Assay

Cell proliferation was analyzed by the Click-iT® Plus EdU Alexa Fluor® 555 Imaging Kit (Invitrogen, cat. C10638) according to the manufacturer's instructions. The HRMCs were co-cultured in transwell plates, and 10 μmol/L EdU was added to the medium for 4 hours. Then, the cells were fixed with 4% paraformaldehyde, permeabilized with 0.2% Triton X-100 and washed. The cells were then incubated at room temperature in the Click-iT® reaction cock- tail for 30 minutes. DAPI was used to dye the nuclei. Cells were observed under a confocal inverted laser

microscope (FV3000, Olympus, Tokyo, Japan). Data were collected from 6 independent experimental repeats.

Enzyme-linked immunosorbent assay

Human plasma was isolated from blood, and the HRMCs medium was collected after stimulated by PDGF-BB (20ng/ml) and TNFα (10ng/ml), while the THP-1 medium was collected after treated by CX3CL1 (50ng/ml) and AZD8797(5mM). The samples were centrifuged (500 g for 10 minutes at 4°C) and stored at −80°C. CX3CL1 level was measured by Human CX3CL1/Fractalkine Quantikine ELISA Kit (DCX310, R&D) according to manufacturer's instructions. MIF was measured by Human MIF (Macrophage Migration Inhibitory Factor) ELISA Kit (E-EL-H6170, elabscience) according to manufacturer's instructions.

Luminex multiplex immunoassay

THP-1 medium was collected after treated by CX3CL1 (50ng/ml) and AZD8797(2mM) and stored at −80°C. Cytokines were measured using a Luminex X-MAP system (Luminex 200 system, Luminex Corporation, Austin, TX, USA) and the Bio-Plex Pro Human Chemokine Panel 40-plex (Bio-Rad Laboratories, California, USA). The assay was conducted according to the manufacturer’s protocol (Wayen Biotechnologies, Shanghai, China). Concentrated human recombinant standards were provided by the vendor and a broad range of standards was used to establish standard curves. The samples and standards tested in this experiment were detected using a Luminex 200 detector, and the fluorescence obtained was automatically calculated and optimized using the software. The original fluorescence of each sample was substituted into the standard curve formula to calculate sample concentration. The experiment was performed with 4 independent biological replicates.

Library construction for RNA-seq and sequencing procedures

Total RNA was isolated using SMART-Seq® HT Kit. Paired-end libraries were synthesized by using the TruSeq® RNA Sample Preparation Kit (Illumina, USA) following TruSeq® RNA Sample Preparation Guide. Briefly, the poly-A containing mRNA molecules were purified using poly-T oligo-attached magnetic beads. Following purification, the mRNA is fragmented into small pieces using divalent cations under 94°C for 8 min. The cleaved RNA fragments are copied into first strand cDNA using reverse transcriptase and random primers. This is followed by second strand cDNA synthesis using DNA Polymerase I and RNase H. These cDNA fragments then go through an end repair process, the addition of a single 'A' base, and then ligation of the adapters. The products are then purified and enriched with PCR to create the final cDNA library. Purified libraries were quantified by Qubit® 2.0 Fluorometer (Life Technologies, USA) and validated by Agilent 2100 bioanalyzer (Agilent Technologies, USA) to confirm the insert size and calculate the mole concentration. Cluster was generated by cBot with the library diluted to 10 pM and then were sequenced on the Illumina HiSeq Xten (Illumina, USA). The library construction and sequencing were performed at Shanghai Biotechnology Corporation.

Supplementary Table 1. Primer sequences for qPCR analysis.

Gene	Nucleotide sequence (5' to 3')	Species
TNFα	F- ggtgaccaactgtcactcatt	human
	R- ccactgaatagtagggcgattac	
IL-6	F- ccctgacccaaccacaaa	human
	R- ggactgcaggaactccttaaa	
IL-1β	F- atgatggcttattacagtggcaa	human
	R- gtcggagattcgtagctgga	
MIF	F- tgcacagcatcggcaagatc	human
	R- aggcgaaggtggagttgttc	
CX3CL1	F- ctcgccaatcccagtgaccttgctc	rat
	R- gattggtagacagcagaactcggccaaatg	
CX3CR1	F- ccatgtgcaagctcacgact	rat
	R- actgtccggttgttcatgga	
TNFα	F- atgggctccctctcatcagt	rat
	R- aaatggcaaatcggctgacg	
IL-6	F- tggagttccgtttctacctg	rat
	R- tagggtttcagtattgctct	
IL-1β	F- gtgctgtctgacccatgtga	rat
	R- ggggaactgtgcagactcaa	
PCNA	F- caatttctagcaacgcctaagat	rat
	R- aagaggaagctgtgtccatagag	
CXCR2	F- cgctgctcatcatgctgttc	rat
	R- gacgaggaccacagcaaaga	

Supplementary Table 2. Cytokine and Chemokine Production by THP-1 activated by CX3CL1 (ng/ml).

	THP1 $_{Control}$	THP1 $_{CX3CL1}$	THP1 $_{CX3CL1+AZD8797}$	P value	P value
CXCL13	0.015±0.030	0.015±0.017	0.000±0.000	> 0.9999	0.3165
CCL24	1.643±0.099	1.563±0.132	1.603±0.150	0.4021	0.6706
CCL26	0.655±0.033	0.688±0.084	0.690±0.106	0.5803	0.9658
CCL11	0.040±0.000	0.040±0.000	0.083±0.061	> 0.9999	0.1237
GM-CSF	0.170±0.012	0.220±0.008	0.185±0.013	0.0001	0.0015
CXCL11	0.693±0.063	0.770±0.071	0.645±0.045	0.1036	0.017
IL-1β	0.00	0.00	0.00	-	-
CXCL8	1.068±0.152	1.165±0.116	1.133±0.056	0.2604	0.6983
IL-10	0.005±0.006	0.018±0.005	0.005±0.006	0.0109	0.0109
IL-16	1.540±0.306	2.648±0.191	1.668±0.145	< 0.0001	0.0002
CXCL10	0.443±0.266	0.833±0.207	0.365±0.266	0.0532	0.0258
CCL2	0.030±0.000	0.030±0.000	0.070±0.014	> 0.9999	< 0.0001
CCL8	0.00	0.005±0.010	0.00	0.2518	0.2518
CCL13	0.025±0.006	0.028±0.017	0.025±0.010	0.7732	0.7732
CCL22	2.550±0.183	2.590±0.257	2.660±0.104	0.7745	0.6178
MIF	882.5±74.23	1205±102.1	924.9±102.5	0.0009	0.0022
CCL3	0.118±0.017	0.115±0.006	0.170±0.035	0.8789	0.0073
CCL15	0.508±0.034	0.510±0.070	0.553±0.069	0.9541	0.3409
CCL20	0.010±0.000	0.010±0.000	0.010±0.000	-	-
CCL23	170.3±4.412	223.6±82.27	197.3±33.25	0.1758	0.4867
CXCL16	3.208±0.139	3.483±0.300	3.568±0.212	0.1200	0.6083
CXCL12	16.72±2.205	16.43±1.231	14.56±1.375	0.8136	0.1452
CCL25	2.043±0.990	2.230±0.556	2.245±0.684	0.7369	0.9785
TNF-α	3.035±0.163	3.270±0.182	2.850±0.147	0.0741	0.0056

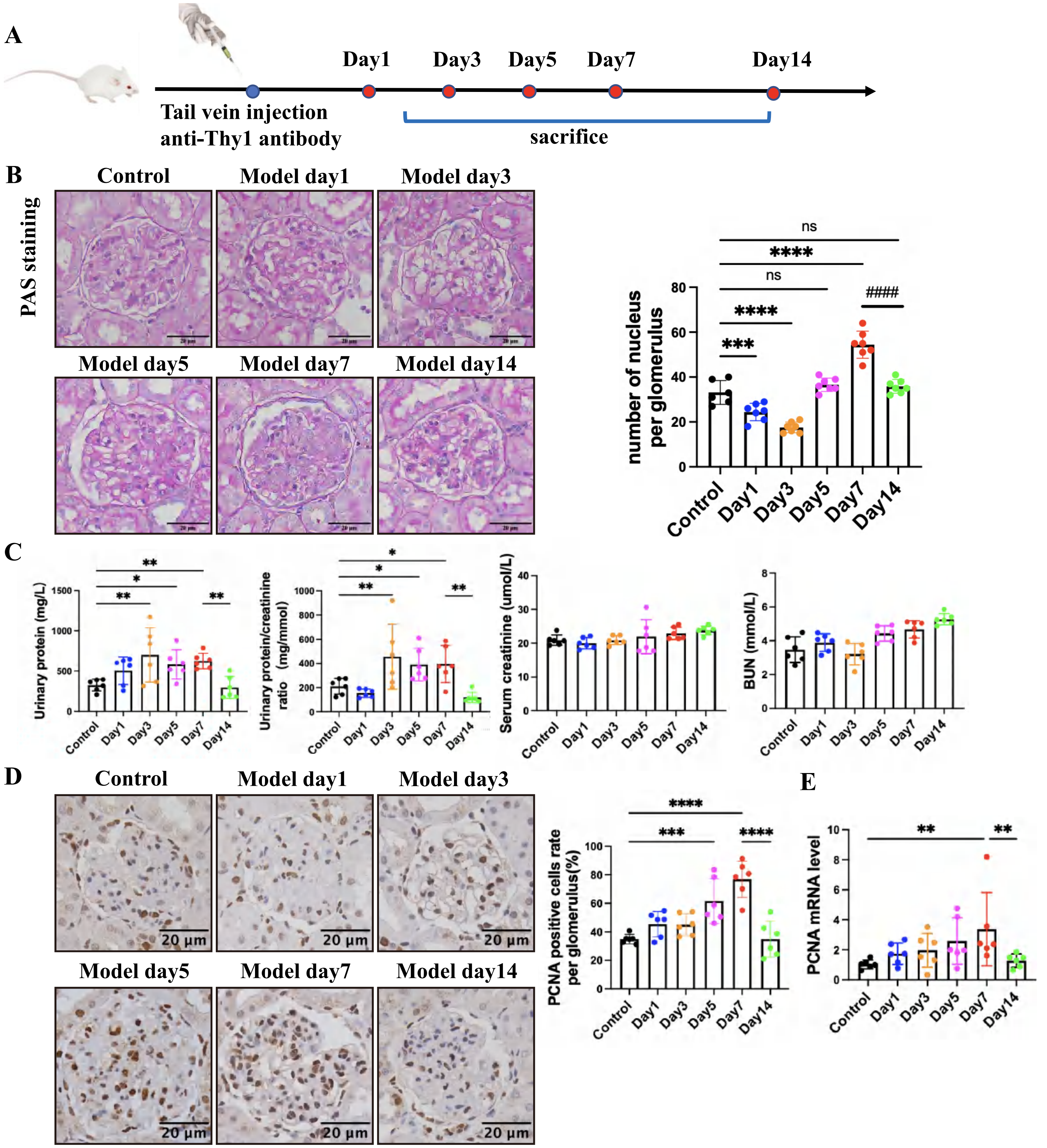

Supplementary Figure 1. Establishment of anti-Thy1 nephritis with different time points.
(A). Experimental outline. Rats have injection of 2.5mg/kg anti-Thy1 antibody via tail vein to establish anti-Thy1 nephritis model. Rats were sacrificed at day1, day3, day5, day7, day14 after model establishment. (B)Representative images and quantitative results of PAS staining of anti-Thy1 nephritis model at different time points. Scale bar, 20um. n=6. (C) The urinary albumin-to-creatinine ratio (UACR), the levels of urinary protein, serum creatinine, blood urea nitrogen (BUN) were determined in indicated groups. n=6. (D and E) Representative images and quantitative results of immunostaining for PCNA and real-time PCR analysis of PCNA mRNA expression of anti-Thy1 nephritis model at different time points.Scale bar, 20um. n=6. Results are presented as the means ± SD, *p<0.05; **p<0.01; ***p<0.001; ****p<0.0001; ns, not significant.

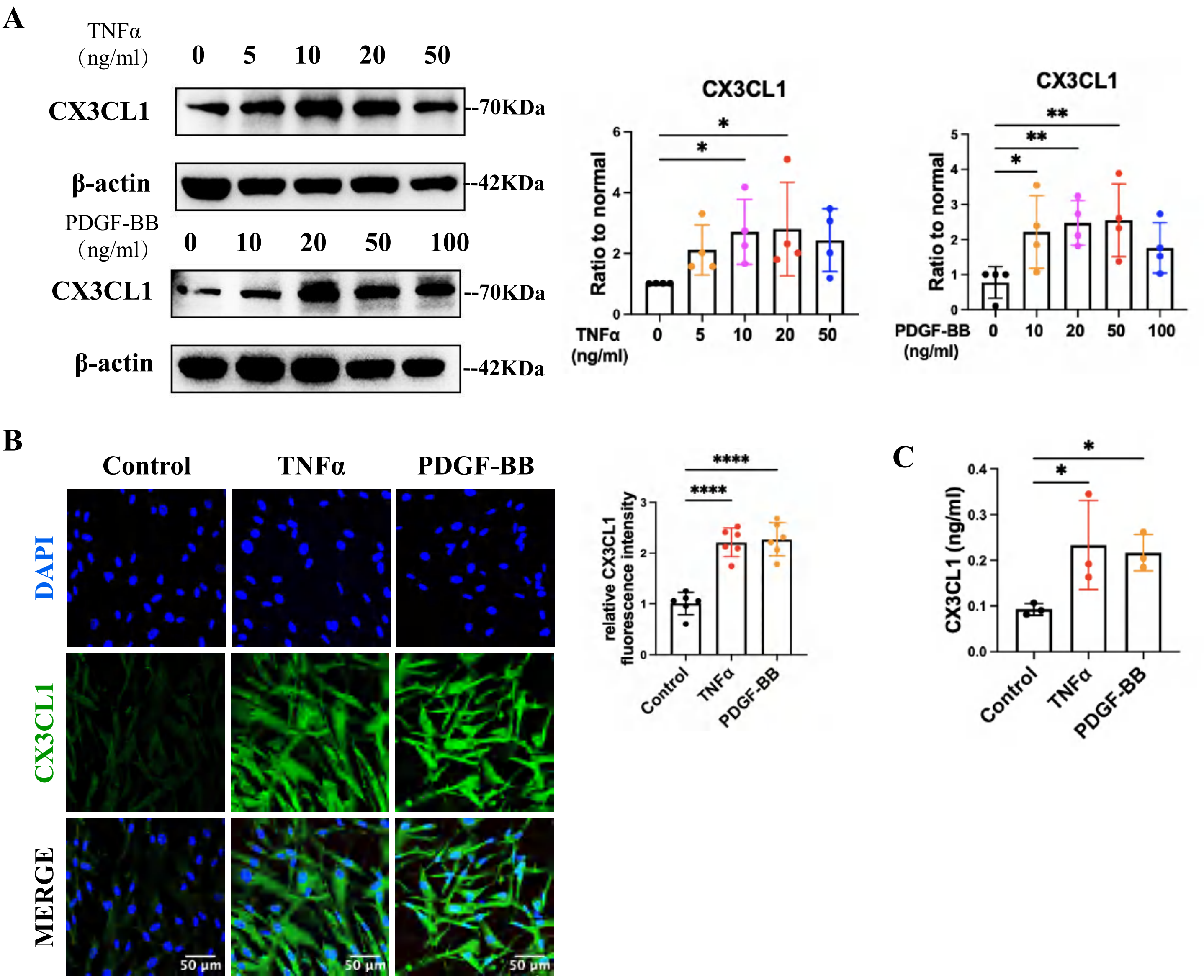

Supplementary Figure 2. The CX3CL1 expression and secrection in HRMCs was up-regulated by TNFα and PDGF-BB.

(A)Western blot analysis of CX3CL1 protein expression levels in HRMCs stimulated by TNFα (0-50ng/ml) and PDGF-BB(0-100ng/ml) for 48 hours. n=4 (B) Immunofluorescence microscopy detection on CX3CL1 expression and (C) ELISA detection on CX3CL1 secretion in HRMCs stimulated by TNFa (10ng/ml) and PDGF-BB (20ng/ml) for 48 hours. n=3-6. Scale bar, 50um. Results are presented as the means (SD), *p<0.05; **p<0.01; ***p<0.001; ****p<0.0001; ns, not significant.   .

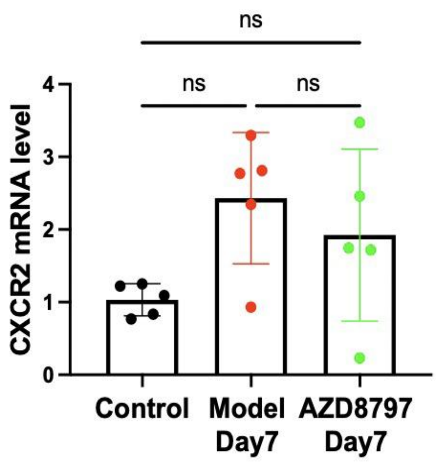

Supplementary Figure 3. Real-time PCR analysis of MsPGN animal model treated with AZD8797.
Real-time PCR analysis of CXCR2 mRNA expression in glomerulus of normal rats and anti-Thy1 nephritis at day7 with or without AZD8797 treatment, n=5. Results are presented as the means ± SD, *p<0.05; **p<0.01; ***p<0.001; ****p<0.0001; ns, not significant.

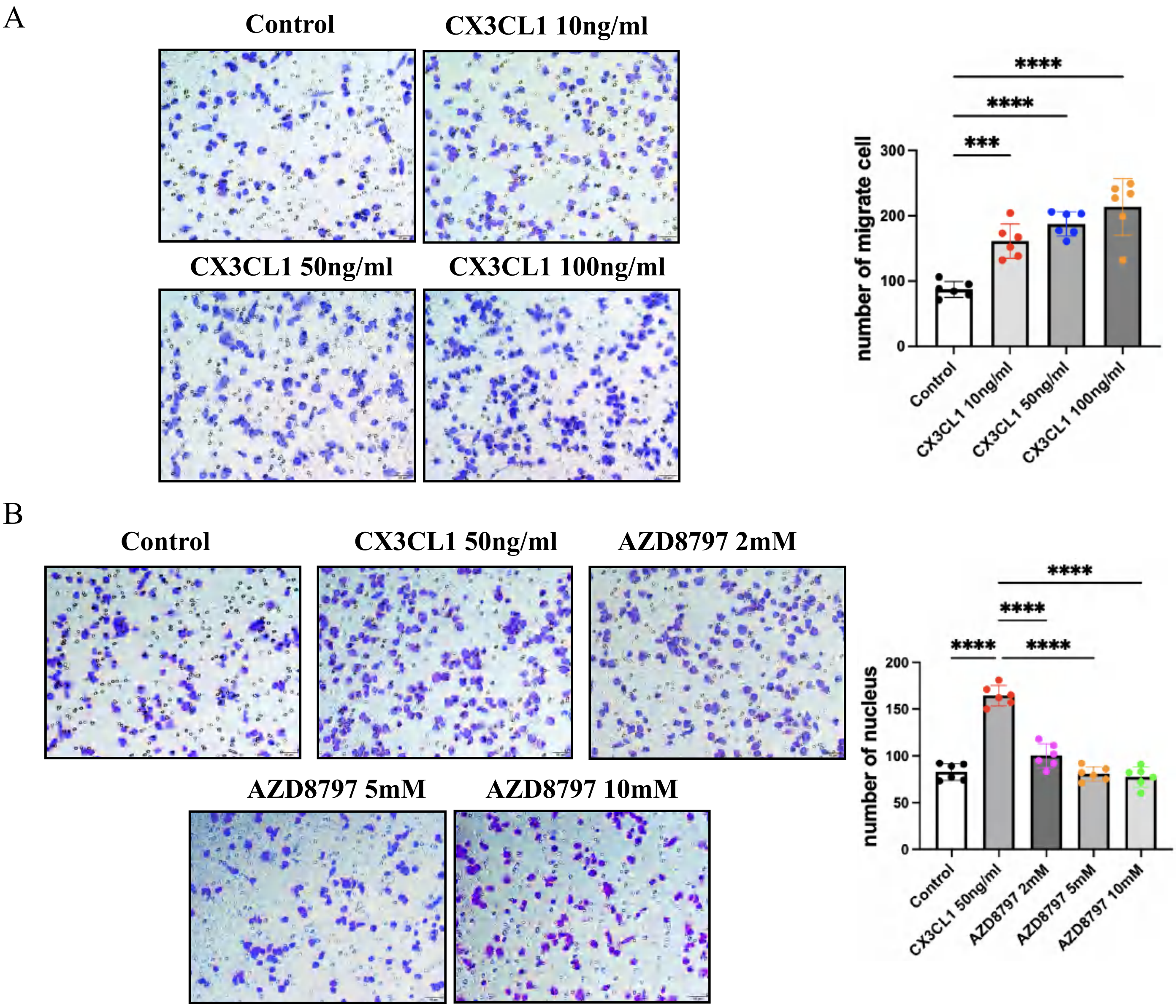

Supplementary Figure 4. Human recombinant protein CX3CL1 promotes THP-1 to migrate, which was suppressed by AZD8797.

(A) Crystal violet staining of THP-1 with treatment of CX3CL1(0-100ng/ml) for 48 hours. n=6. Scale bar, 50um. (B) Crystal violet staining of THP-1 with treatment of CX3CL1(50ng/ml) and AZD8797(0-10mM) for 48 hours. n=6. Scale bar, 50um. Results are presented as the means ± SD, *p<0.05; **p<0.01; ***p<0.001; ****p<0.0001; ns, not significant.

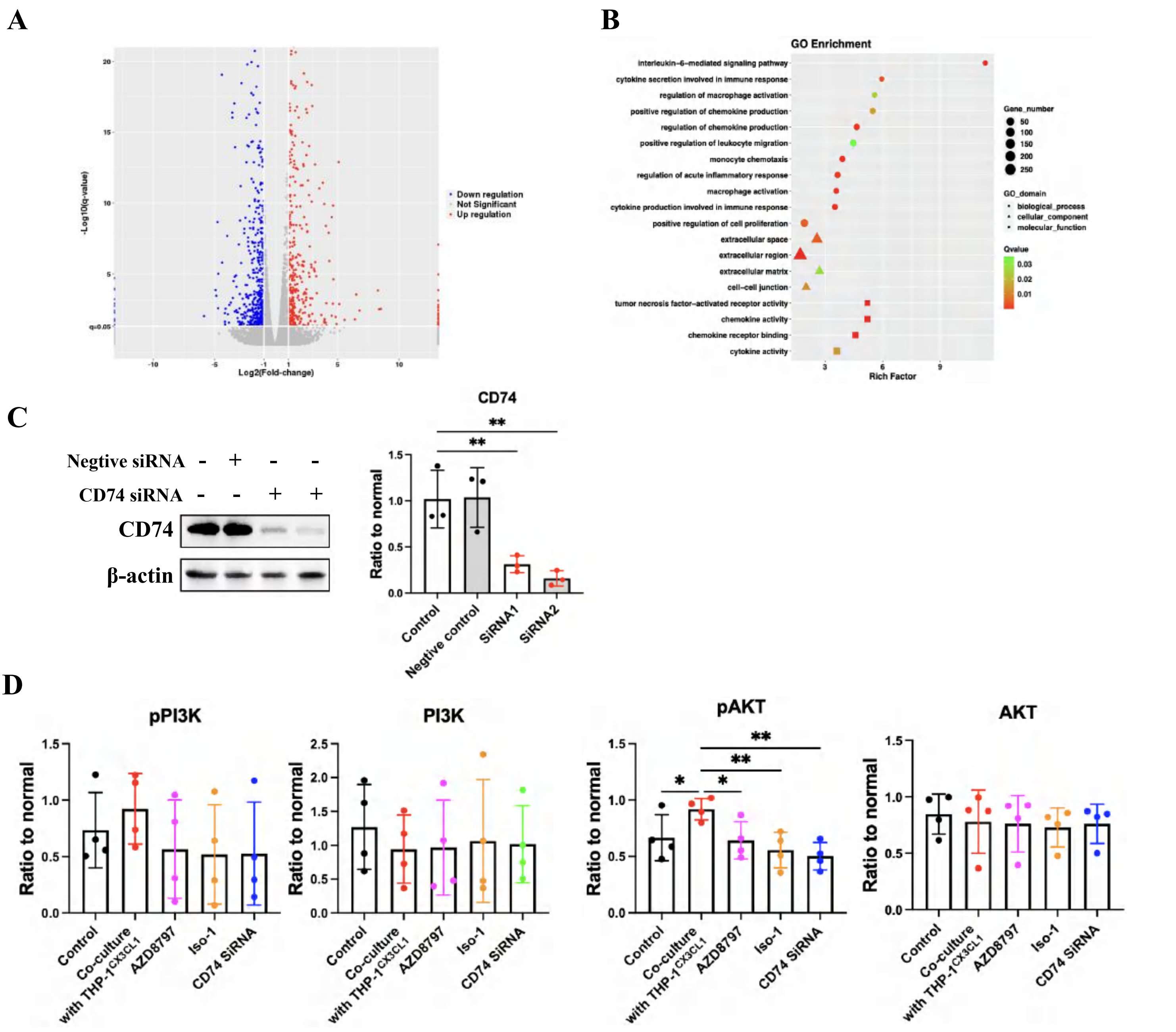

Supplementary Figure 5. RNA-seq analysis and WB analysis of CD74 and PI3K/AKT.
(A) The volcano plot of Gene expression profiling by RNA-seq. (B) GO analysis by RNA-seq. (C) Western blotting detects the transfection efficiency of CD74 siRNA. n=3. (D) Western blot analysis of pAKT, AKT, pPI3K, PI3K expression in normal HRMCs, HRMCs co-cultured with THP-1^{CX3CL1} for 48 hours treated with AZD8797, ISO-1 or not, and CD74 knockdown HRMCs co-cultured with THP-1^{CX3CL1} for 48 hours, n=4. Results are presented as the means ± SD, *p<0.05; **p<0.01; ***p<0.001; ****p<0.0001; ns, not significant.

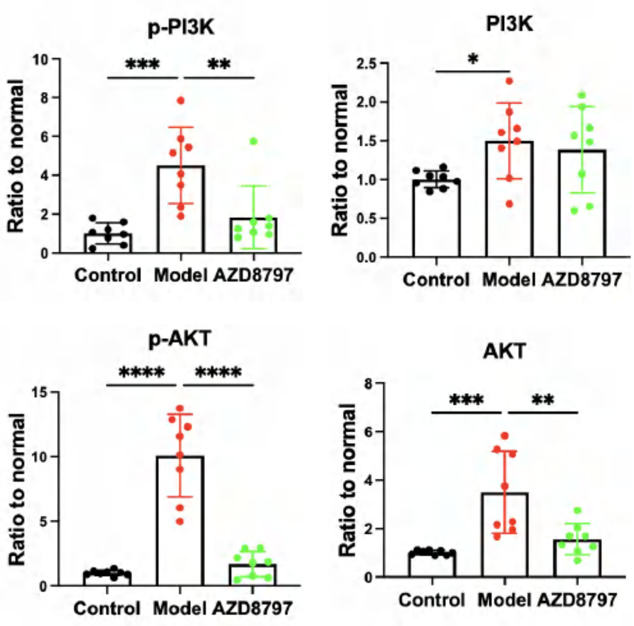

Supplementary Figure 6. WB analysis of MsPGN animal model treated with AZD8797.

Western blot analysis of pAKT, AKT, pPI3K, PI3K expression level in glomerulus of normal rats and anti-Thy1 nephritis at day7 with or without AZD8797 treatment. n=6. Results are presented as the means ± SD, *p<0.05; **p<0.01; ***p<0.001; ****p<0.0001; ns, not significant.

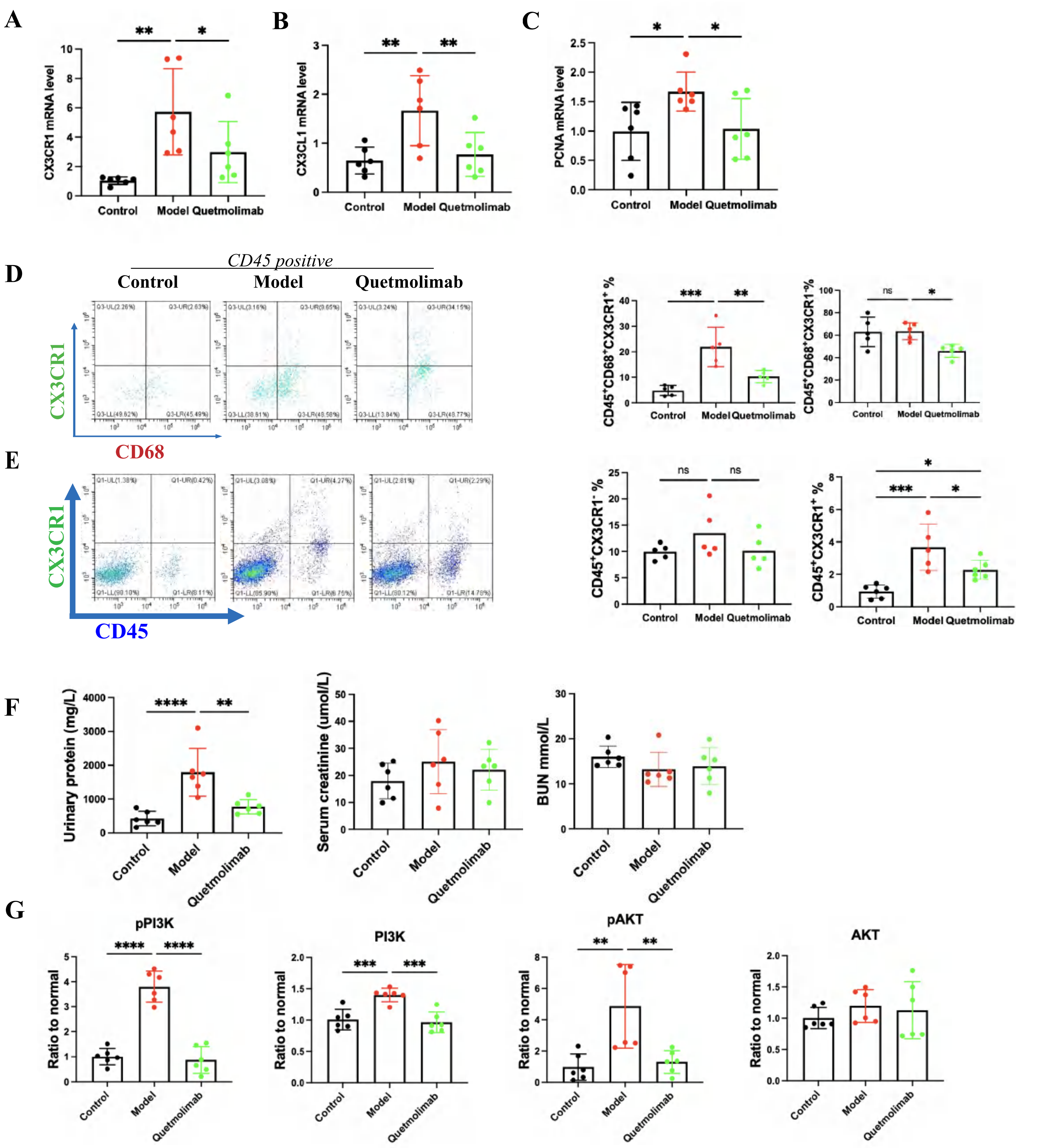

Supplementary Figure 7. Real-time PCR analysis, biochemical criterion and flow cytometry analysis of anti-Thy1 nephritis treated with quetmolimab.

(A,B,C) Real-time PCR analysis of CX3CR1, CX3CL1 and PCNA mRNA expression in indication groups. n=6. (D) Flow cytometry analyses of $CD45^+CD68^+CX3CR1^+$cells and $CD45^+CD68^+CX3CR1^-$cells in glomerulus of indication groups. n=5. (E) Flow cytometry analyses of $CD45^+CX3CR1^+$, $CD45^+CX3CR1^-$ cells in glomerulus of indication groups. n=5.(F) The levels of urinary protein, serum creatinine, blood urea nitrogen (BUN) was determined in indicated groups. n=6. (G) Western blot analysis of pAKT, AKT, pPI3K, PI3K expression level in glomerulus of normal rats and anti-Thy1 nephritis at day7 with or without quetmolimab treatment. n=6. Results are presented as the means ± SD, *p<0.05; **p<0.01; ***p<0.001; ****p<0.0001; ns, not significant.
